# Design, Synthesis and Anticancer Activity of 6-Substituted-1-(3,4,5-trimethoxyphenyl)-*1H*-indole Against Tubulin Polymerisation

**DOI:** 10.3390/molecules30234538

**Published:** 2025-11-24

**Authors:** Yuanna Gu, Conghao Gai, Sijie Zou, Yan Song, Juan Zhang, Qingjie Zhao, Xiaoyun Chai, Peipei Wang

**Affiliations:** 1 College of Food Science and Technology, Shanghai Ocean University, Shanghai 201306, China; giwianna0604@163.com; 2 Department of Organic Chemistry, School of Pharmacy, Naval Medical University, Shanghai 200433, China; c.gai2@outlook.com; 3 Department of Pharmacy, PLA Naval Medical Center, Naval Medical University, Shanghai 200052, China; 18755222267@163.com (S.Z.); songyan455@126.com (Y.S.); lyzj0706@163.com (J.Z.); 4 Marine Biomedical Science and Technology Innovation Platform of Lin-Gang Special Area, Shanghai 201306, China

**Keywords:** fragment-based drug discovery, tubulin polymerization inhibitors, antitumor activity, cell cycle arrest, indole derivatives

## Abstract

Using virtual FragLites screening and a fragment-based drug discovery (FBDD) strategy, we designed and synthesized a series of 6-substituted-1-(3,4,5-trimethoxyphe-nyl)-*1H*-indole derivatives as potential tubulin polymerization inhibitors. Among them, compound **3g** exhibited the best antiproliferative activity within this series and affected microtubule dynamics in a concentration-dependent manner. Further studies indicated that **3g** induced G_2_/M cell-cycle arrest and triggered apoptosis in MCF-7 cells. In vivo, **3g** achieved tumor growth inhibition rates of 23.3% and 44.2% at 20 mg/kg and 50 mg/kg, respectively, without evident systemic toxicity. These results suggest that **3g** shows preliminary antitumor efficacy and may serve as a starting point for further mechanistic and structural studies. Further optimization and detailed pharmacokinetic and toxicity studies are merited to advance these inhibitors in preclinical development.

## 1. Introduction

Tubulin polymerizes into a dynamic microtubule network via *α/β*-tubulin heterodimers, which play essential roles in cell division, migration, and signal transduction of the eukaryotic cytoskeleton [1,2]. Microtubules form the spindle apparatus to mediate chromosome segregation during mitosis [3]. Dysregulated tubulin is closely associated with malignant progression in cancer cells [4,5,6]. Due to dysregulated cell cycle control, cancer cells display uncontrolled proliferation and genomic instability, leading to an increased dependence on microtubule function [7,8].

Microtubule-targeting agents (MTAs) interfere with microtubule dynamics by inhibiting either polymerization or depolymerization, thereby blocking mitosis in cancer cells and inducing G_2_/M phase arrest and apoptosis [9,10,11]. MTAs are typically classified into two classes: microtubule-stabilizing agents such as taxanes and microtubule-destabilizing agents, including colchicine and combretastatin A-4 [12,13,14]. The former stabilize microtubules by binding to the taxane site on *β*-tubulin, whereas the latter inhibit microtubule assembly by occupying the colchicine-binding site (CBS), a well-characterized allosteric pocket on *β*-tubulin [11,12]. However, the clinical efficacy of existing MTAs is limited by drug resistance, primarily due to P-glycoprotein (P-gp) overexpression leading to enhanced drug efflux, or *β*-tubulin mutations that reduce drug-binding affinity [15,16,17,18]. Consequently, the development of colchicine-binding site inhibitors (CBSIs) has emerged as a promising strategy for overcoming drug resistance [12,19,20]. Fragment-based drug discovery (FBDD) has emerged as a powerful strategy in early-stage lead identification [21,22]. FragLites is an innovative and highly effective technique in this field [23]. It employs small halogenated fragments (e.g., Cl and Br) and leverages their anomalous scattering signals in X-ray crystallography to rapidly identify potential binding sites on the protein surface, followed by structural optimization through fragment linking or growth [24,25,26]. In this study, guided by virtual FragLites screening and the FBDD strategy, we designed and synthesized a series of 6-substituted-1-(3,4,5-trimethoxyphenyl)-*1H*-indole derivatives as potential tubulin polymerization inhibitors. Although the 3,4,5-trimethoxyphenyl–indole scaffold is a known pharmacophore in CBSIs [27,28,29], our work focuses on optimizing substitution patterns to evaluate their preliminary antitumor activities both in vitro and in vivo. This work provides new insight into the structural optimization of 3,4,5-trimethoxyphenyl–indole CBSIs and lays the groundwork for the development of improved microtubule inhibitors.

## 2. Results and Discussion

### 2.1. Chemistry

**Figure 1:**
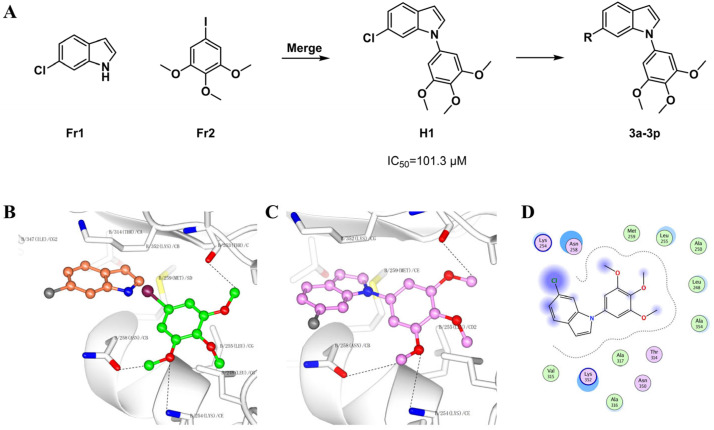
(**A**) Design rationale for the hit compound (**H1**) and its optimized derivatives (**3a**–**3p**). (**B**) Predicted binding pose of two fragments 6-chloro-*1H*-indole (**Fr1**) in orange and 5-iodo-1,2,3-trimethoxybenzene (**Fr2**) in green, respectively, within the colchicine-binding site of *β*-tubulin (PDB ID: 3E22). (**C**) Predicted binding pose of the merged hit compound **H1** in pink at the colchicine-binding site. (**D**) Two-dimensional schematic of the detailed interactions between hit **H1** and the colchicine-binding pocket. The computational docking was achieved by MOE. The image was generated by CCP4MG 2.10.6.

Initially, a virtual fragment library was employed to explore potential ligandable sites on *β*-tubulin, particularly around the nucleotide-binding cleft. Preliminary molecular docking (Figure 1B) identified two fragment hits, 6-chloro-*1H*-indole and 5-iodo-1,2,3-trimethoxybenzene, both exhibiting favorable binding conformations within this region (PDB ID:3E22).

The trimethoxybenzene fragment established hydrogen-bonding interactions with Asn101 and Lys252, while the indole fragment (**Fr1**) fitted well into a nearby hydrophobic pocket composed of Lys352 and Ile347. Interestingly, the spatial complementarity between the iodine atom of the trimethoxybenzene fragment and the NH group of the indole scaffold suggested a feasible fragment-merging strategy. Guided by this model, the merged compound exhibited a measurable inhibitory effect on tubulin polymerization with an IC_50_ value of 101.3 μM, validating the design concept.

In the predicted binding pose, the 6-chloro substituent of the indole ring was solvent-exposed, indicating that further modification at this position would be structurally tolerated. Therefore, a series of 6-substituted derivatives (**3a**–**3p**) was designed and synthesized, aiming to optimize the binding affinity for the colchicine-binding pocket and evaluate its anti-tubulin inhibitory activities by interfering with microtubule dynamics.

**Scheme 1:**
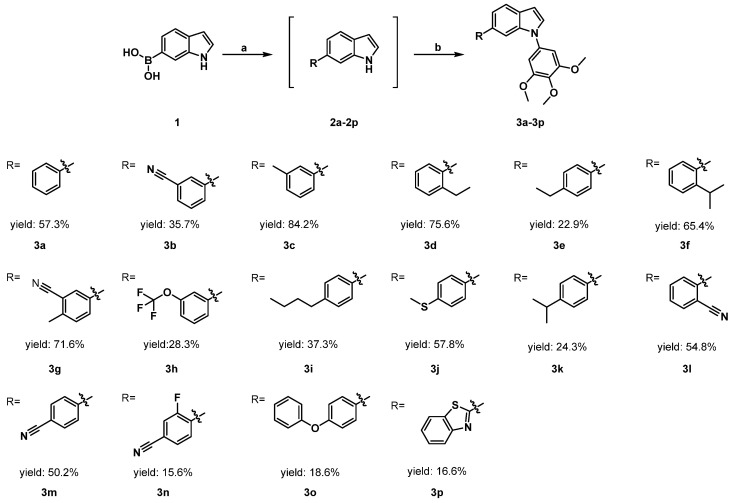
Synthesis of 6-substituted-1-(3,4,5-trimethoxyphenyl)-*1H*-indole species. Reaction condition: (**a**) iodobenzene analogs, Pd(PPh_3_)_4_ (0.06 eq.), Na_2_CO_3_ (2 eq.), 1,4-Dioxane, H_2_O, 80 °C, and overnight; (**b**) 5-iodo-1,2,3-trimethonxybenzene, CuI (0.15 eq.), Cs_2_CO_3_ (0.6 eq.), DMF, 150 °C, and 16 h.

The synthetic route to the target compounds **3a**–**3p** is outlined in Scheme 1. The synthesis commenced with a Suzuki–Miyaura cross-coupling reaction between 6-substituted indole boronic acids and 5-iodo-1,2,3-trimethoxybenzene. The crude biaryl intermediates **2** were used directly in the subsequent step without further purification. Then, the Ullmann-type N-arylation was employed to form a new C-N bond, which constructed the N1-substituted indole scaffold, affording the desired 6-substituted-1-(3,4,5-trimethoxyphenyl)-*1H-*indole derivatives (**3a**–**3p**) in decent yields. All final compounds were characterized by ^1^H NMR, ^13^C NMR, and HRMS, and their structures were consistent with the expected molecular frameworks.

### 2.2. Biological Evaluation

#### 2.2.1. Binding Affinity to Tubulin Protein

To detect the direct interaction between the synthesized compounds and tubulin, biolayer interferometry (BLI) experiments were performed using purified porcine brain tubulin immobilized on Super Streptavidin (SSA) biosensor [30]. Compounds **3a**–**3p** were tested at concentrations ranging from 0 to 200 μM. Association and dissociation kinetics were monitored in real time, and equilibrium dissociation constants (K_D_) were determined by global fitting of the sensorgrams to a 1:1 binding model. All measurements were conducted in triplicate, and results are reported as mean ± SD.

Among the tested derivatives, compound **3g**, bearing a 3-cyano-4-methyl substituent at the 6-position of the indole ring, exhibited the strongest binding affinity toward tubulin, with a K_D_ value of 13 μM. In contrast, most other compounds displayed moderate binding with K_D_ values in the range of 20–50 μM (e.g., **3a**, **3b**, **3c**, **3d**, **3f**, **3l**, **3o**), or undetectable binding (e.g., **3h**, **3i**, **3j**, **3k**, **3m**, **3p**) under the tested conditions (Table 1). These results validated tubulin as the primary molecular target of **3g**.

The binding affinity analysis provides direct biophysical evidence that compound **3g** interacts with tubulin at sub-micromolar levels, supporting its mechanism of action as a microtubule-targeting agent.

#### 2.2.2. Antiproliferative Activity Against Cancer Cell Lines

To evaluate the anticancer potential of the synthesized compounds, a panel of six human and murine cancer cell lines representing different tissue origins was selected, including MCF-7 (human breast adenocarcinoma), MDA-MB-231 (human triple-negative breast cancer), A549 (human non-small cell lung cancer), HeLa (human cervical cancer), A375 (human melanoma), and B16-F10 (murine melanoma) (Table 2). The antiproliferative activity of compounds **3a**–**3p** was assessed using the standard CCK-8 assay after 48 h of drug treatment at varying concentrations. DMSO was used as the vehicle control, and all experiments were performed in triplicate to ensure reproducibility.

Among the tested derivatives, compound **3g** also exhibited the most potent and broad-spectrum antiproliferative activity across all six cell lines. The IC_50_ values of **3g** were determined to be 2.94 ± 0.56 μM (MCF-7), 1.61 ± 0.004 μM (MDA-MB-231), 6.30 ± 0.30 μM (A549), 6.10 ± 0.31 μM (HeLa), 0.57 ± 0.01 μM (A375), and 1.69 ± 0.41 μM (B16-F10), respectively, indicating sub-micromolar to low micromolar efficacy in multiple cancer types.

These results highlight compound **3g** as a promising lead candidate for further development as a tubulin-targeting anticancer agent with potential application in both breast cancer and melanoma-derived malignancies.

#### 2.2.3. Inhibition of Tubulin Polymerization

**Figure 2:**
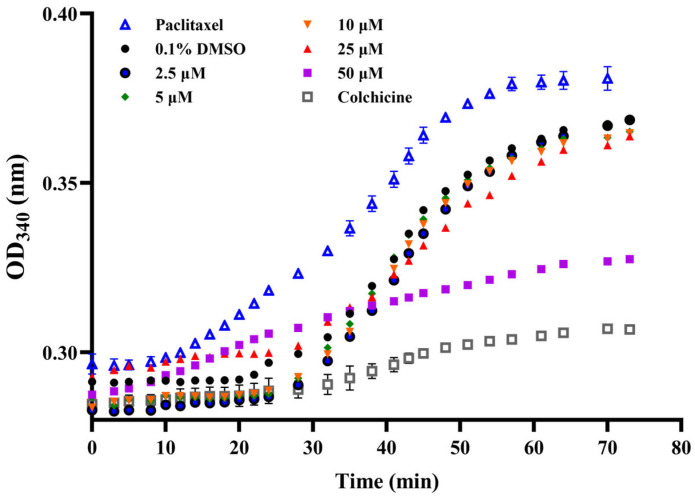
Compound **3g** disturbs microtubule polymerization. Tubulin polymerization assay with 2.5, 5, 10, 25, and 50 μM of compound **3g**; 0.1% DMSO; 10 μM paclitaxel; and 10 μM colchicine. Absorbance at 340 nm was determined every 1 min.

The ability of compound **3g** to disrupt microtubule dynamics was assessed using an in vitro tubulin polymerization assay [31]. As shown in Figure 2, compound **3g** inhibited tubulin polymerization in a concentration-dependent manner over the range of 2.5–50 μM. At 50 μM, **3g** markedly suppressed tubulin assembly, indicating potent inhibition at higher concentrations. As expected, colchicine and paclitaxel exhibited characteristic depolymerizing and stabilizing effects, respectively, while 0.1% DMSO had no significant influence on polymerization. These findings confirm that compound **3g** effectively interferes with tubulin polymerization in vitro, supporting its proposed mechanism of action as a microtubule-targeting agent.

#### 2.2.4. Inhibition of Cancer Cell Colony Formation

**Figure 3:**
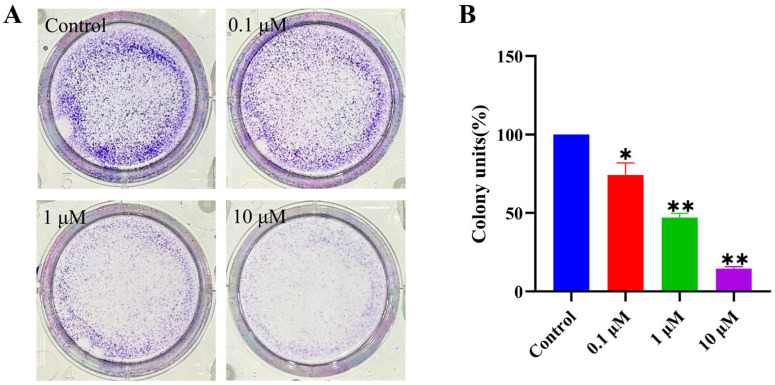
Inhibition of MCF-7 Cell Colony Formation by Compound **3g**. (**A**) **3g** inhibited the colony formation of MCF-7 cells at different concentrations in a dose-dependent manner. (**B**) Colony units in the different groups after treatment with **3g**. Number of colonies was counted after treating MCF-7 cells with the indicated concentration of compound **3g** for 24 h (n = 3, * *p* < 0.05, ** *p* < 0.01 vs. DMSO control, one-way ANOVA).

To assess the long-term antiproliferative potential of compound **3g**, a colony formation assay was conducted using MCF-7 breast cancer cells. Treatment with compound **3g** for 24 h significantly suppressed colony-forming capacity in a dose-dependent manner (Figure 3). Notably, low concentrations (0.1 μM) led to a moderate reduction in clonogenic survival, while higher doses (1 μM and 10 μM) produced progressively greater inhibition, culminating in near-complete suppression of colony formation at 10 μM. These results indicate that compound **3g** effectively impairs the self-renewal and long-term survival of cancer cells, supporting its potential as a potent antiproliferative agent.

#### 2.2.5. Suppression of MCF-7 Cell Migration

**Figure 4:**
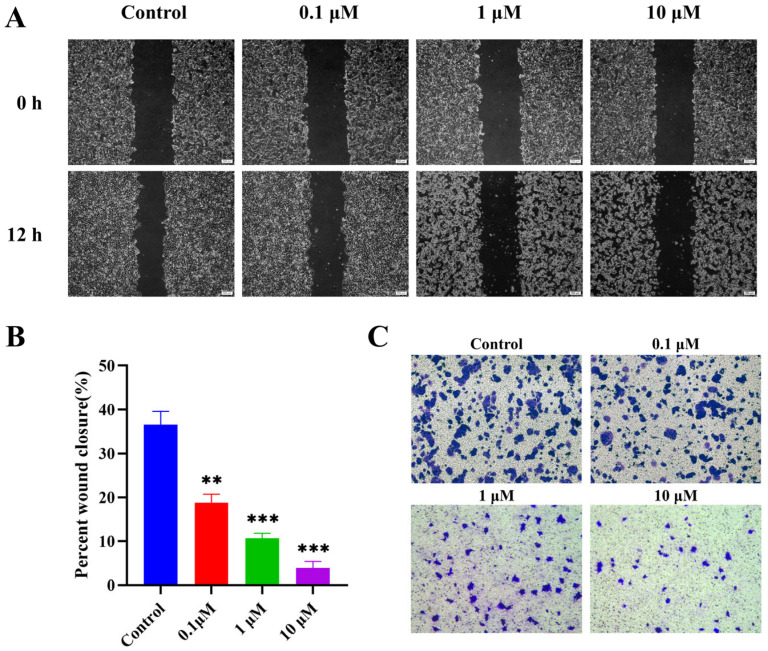
Effect of compound **3g** on the migration of MCF-7 breast cancer cells. (**A**) **3g** inhibited the migration of MCF-7 cells at different concentrations in a dose-dependent manner. Representative images of wound healing were captured by an inverted microscope at 0 h and 24 h treatment. Scale bar = 200 μm. (**B**) Wound closure percentages in the different groups after treatment with **3g** for 24 h. (**C**) Results of the transwell migration assay. Representative images of the stained migrated cells after incubation with **3g** at different concentrations for 48 h. Scale bar = 200 μm. Data are presented as the mean ± SD of three independent experiments. Bar values represent the standard deviation SD. ** *p* < 0.01, and *** *p* < 0.001 (one-way ANOVA).

The impact of compound **3g** on MCF-7 cell migration was assessed using wound healing and transwell migration assays. As shown in Figure 4A, treatment with **3g** markedly inhibited wound closure compared to the untreated control, indicating impaired lateral motility. In parallel, the transwell migration assay further confirmed the antimigratory effect of **3g**, with a significant reduction in the number of cells traversing the membrane after 24 h exposure (Figure 4C). Both assays consistently demonstrated that compound **3g** suppressed the migratory capacity of MCF-7 cells in a dose-dependent manner.

#### 2.2.6. Antimicrotubule Activity in MCF-7 Cells

**Figure 5:**
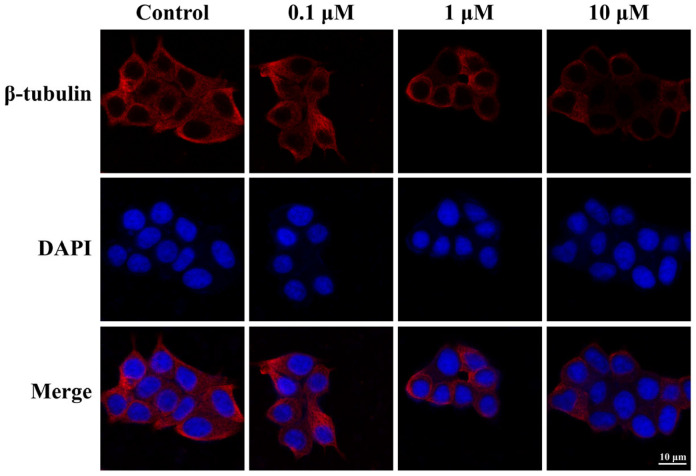
Effects of compound **3g** on microtubules. MCF-7 cells were treated with vehicle control 0.1% DMSO, compound **3g** (0.1μM, 1μM, and 10 μM for 24 h). Microtubules were visualized with an anti-*β*-tubulin antibody (red), and the cell nucleus was visualized with DAPI (blue).

Given the potent in vitro antiproliferative activity of compound **3g**, its effects on microtubule organization were further investigated using immunofluorescence staining in MCF-7 cells. The results are shown in Figure 5. Untreated control cells exhibited an intact and well-organized microtubule network, with fine, fibrous microtubules (red) distributed throughout the cytoplasm and surrounding the nucleus (blue). In contrast, treatment with compound **3g** at concentrations of 0.1, 1, and 10 μM for 24 h resulted in progressive disruption of the microtubule architecture. The network appeared disassembled and fragmented in a dose-dependent manner, indicating significant cytoskeletal destabilization. Compound **3g** potently suppressed tubulin polymerization in a concentration-dependent manner. These results, together with the immunofluorescence data, strongly suggest that compound **3g** exerts its antiproliferative effects through microtubule destabilization and disruption of cytoskeletal integrity in cancer cells.

#### 2.2.7. Analysis of Cell Cycle Analysis

**Figure 6:**
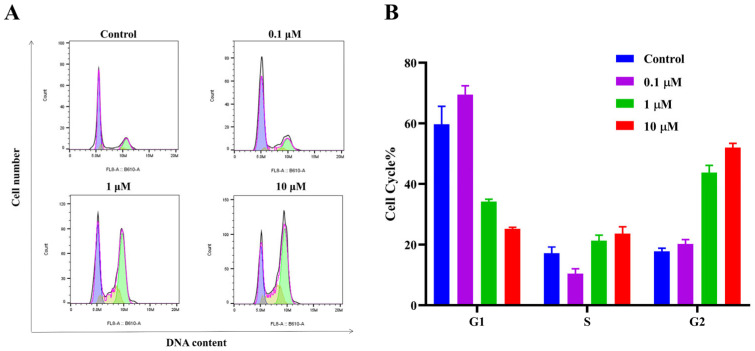
Cell cycle arrest induced by compound **3g**. (**A**) Compound **3g** induced G_2_/M arrest in MCF-7 cells. MCF-7 cells were incubated with varying concentrations of compound **3g** (0.1, 1, and 10 μM) for 48 h. The percentages of cells in different phases of the cell cycle were analyzed by FlowJo 10.8.1. (**B**) Histograms display the percentage of cell cycle distribution after treatment with compound **3g**. Data are expressed as mean ± standard deviation from three independent experiments. Statistical significance was determined by one-way ANOVA followed by Tukey’s post hoc test.

Given that microtubule polymerization inhibitors are known to disrupt mitosis and induce cell cycle arrest, we evaluated the effect of compound **3g** on cell cycle progression in MCF-7 cells by flow cytometry. As shown in Figure 6A,B, treatment with **3g** (10 μM, 48 h) resulted in a marked accumulation of cells in the G_2_/M phase, indicating that **3g** effectively induces cell cycle arrest at this checkpoint.

#### 2.2.8. Analysis of Cancer Cell Apoptosis Analysis

**Figure 7:**
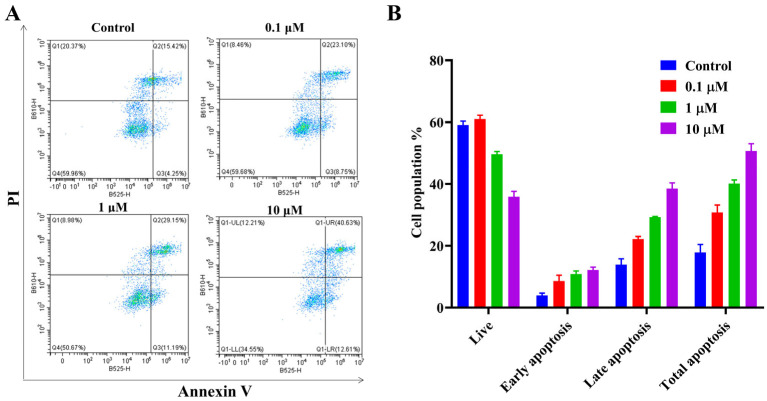
Effects of compound **3g** on apoptosis in MCF-7 cells. (**A**) Apoptosis induction by compound **3g** in MCF-7 cells. Cells were treated with increasing concentrations of **3g** (0.1, 1, and 10 μM) for 48 h, followed by Annexin V-FITC/PI staining and flow cytometric analysis. Cell populations were categorized as necrotic (**upper left quadrant**), late apoptotic (**upper right**), viable (**lower left**), and early apoptotic (**lower right**). (**B**) Quantification of apoptotic populations. Bar graphs show the percentage of cells in each apoptotic stage after treatment. Data are presented as mean ± SD from three independent experiments. Statistical significance was determined by one-way ANOVA followed by Tukey’s post hoc test.

Given that mitotic arrest induced by tubulin polymerization inhibitors is commonly associated with apoptosis, an Annexin V-FITC/propidium iodide (PI) assay was conducted to evaluate the pro-apoptotic effect of compound **3g** in MCF-7 cells. Treatment with increasing concentrations of **3g** (0.1, 1, and 10 μM) for 48 h led to a dose-dependent increase in apoptotic cell populations, with 10 μM **3g** significantly inducing apoptosis in MCF-7 cells (Figure 7A,B).

#### 2.2.9. In Vivo Antitumor Efficacy

**Figure 8:**
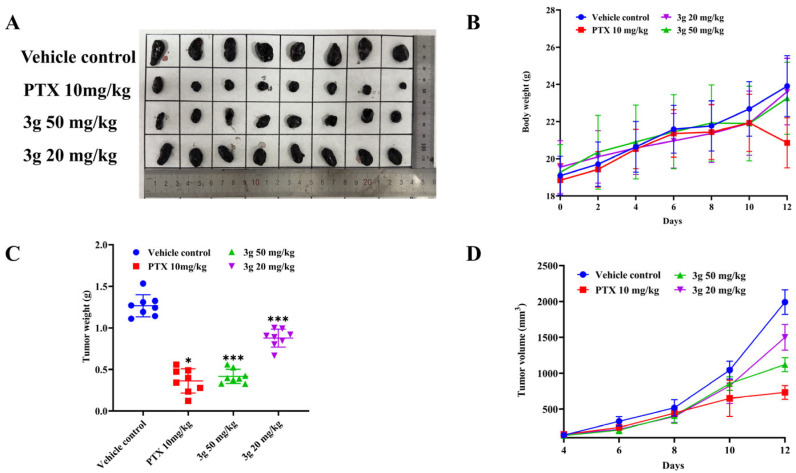
Compound **3g** inhibits melanoma tumor growth in vivo. Murine melanoma B16-F10 xenograft tumors were established in mice, which were then administered vehicle, PTX (10 mg/kg per day), compound **3g** (20 mg/kg per day), and compound **3g** (50 mg/kg per day) for 14 days. The mice were sacrificed, and the tumors were weighed. (**A**) Images of tumors from mice at 14 days after initiation of treatment. (**B**) Body weight changes in mice during treatment. (**C**) Weight of the excised tumors of each group. * *p* < 0.05, and *** *p* < 0.001 vs. control group. (**D**) Tumor volume changes in mice during treatment. Statistical analyses were performed using one-way ANOVA (tumor weight) or two-way repeated-measures ANOVA (tumor volume).

The in vivo antitumor activity of compound **3g** was evaluated using a mouse melanoma tumor model in BALB/c mice. Tumors were established by subcutaneous inoculation of B16F10 cells, and treatment was initiated when tumors reached a measurable size. Mice were administered vehicle control, paclitaxel (PTX, 10 mg/kg), or compound **3g** (20 and 50 mg/kg) via intraperitoneal injection once daily for 14 consecutive days. As shown in Figure 8A, compound **3g** significantly inhibited tumor growth in a dose-dependent manner, achieving tumor growth inhibition (TGI) rates of 23.34% and 44.18% at 20 and 50 mg/kg, respectively, compared to 59.84% TGI with PTX at 10 mg/kg.

**Figure 9:**
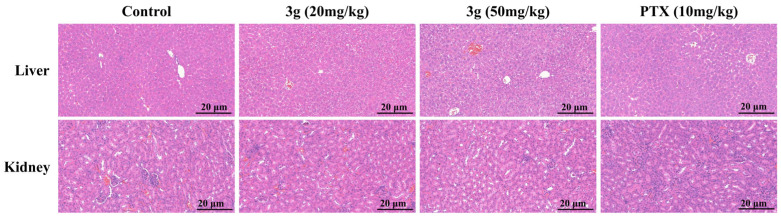
Pathological sections of major tissues (liver and kidney) were obtained from mice bearing melanoma tumors. Organs were stained with H&E, and the representative images were captured.

Importantly, treatment with compound **3g** did not induce significant body weight loss throughout the study period (Figure 8B), suggesting good systemic tolerability. In addition, histopathological analysis of major organs by hematoxylin and eosin (H&E) staining did not reveal obvious morphological abnormalities in the liver or kidneys of treated animals (Figure 9). As no serum biochemical analyses were performed, these results should be interpreted as preliminary indications of tolerability based solely on histological assessment.

## 3. Materials and Methods

### 3.1. General Chemistry Methods

Unless otherwise specified, all chemicals and reagents were obtained from commercial suppliers and were used without further purification. ^1^H and ^13^C NMR spectra were recorded on a Bruker Avance (Billerica, MA, USA) at 500 MHz, using CDCl_3_ or DMSO-*d6* as solvent and tetramethylsilane as internal standard. Chemical shifts are reported in parts per million (ppm). Multiplicities are reported as follows: singlet (s), doublet (d), triplet (t), quartet (q), double doublet (dd), and multiplet (m). Compound purities were estimated by reversed-phase C18 high-performance liquid chromatography, with UV detection at 254 nm. The major peak area of each tested compound was ≥95% of the combined total peak area. High-resolution mass spectra (HRMS) were measured with an Agilent Technologies 6538 UHD accurate-Mass Q-TOF MS spectrometer (Santa Clara, CA, USA) using ESI. Column chromatography was carried out using commercially available silica gel (200–300 mesh) under pressure. Flash column chromatography was performed on silica gel (60 Å, 200–300 mesh) under positive pressure. Elution was carried out using petroleum ether/ethyl acetate mixtures (from 50:1 to 5:1, *v*/*v*) as the mobile phase unless otherwise stated.

### 3.2. Docking Studies

The molecular modeling studies of the household Virtual FragLites library were carried out using Molecular Operating Environment MOE version 2019.01. The crystal structure of tubulin (PDB ID: 3E22) complex was chosen for the docking study, which was downloaded from the protein data bank website (http://www.rcsb.org). The protein structures were prepared after downloading using the MOE quick preparation tool. The compound data was prepared by minimizing energy, adding hydrogen atoms, calculating partial charges and calculating potential energy. The GBVI/WSA ΔG is a force field-based scoring function that estimates the free energy of binding of the ligand from a given pose. Ligand poses were ranked according to their binding free energy. Figures were prepared using the CCP4MG 2.10.6 suite.

### 3.3. Synthesis of 6-Phenyl-1H-indoles (Intermediate 1)

In a 100 mL round-bottom flask containing indole-6-boronic acid (1 eq.), iodobenzenes (1 eq.), Pd(PPh_3_)_4_ (0.06 eq.), and Na_2_CO_3_ (2 eq.) were added to 1,4-dioxane (8 mL) and water (4 mL). The reaction mixture was stirred at 80 °C for 12 h under an argon atmosphere. Reaction progress was monitored by TLC. The mixture was cooled to room temperature, diluted with water, and extracted with ethyl acetate (3 × 20 mL). The combined organic layers were dried over anhydrous Na_2_SO_4_, filtered, and concentrated in vacuo, which afforded the crude intermediates **2a**–**2p**.

### 3.4. Synthesis of 6-Phenyl-1-(3,4,5-trimethoxyphenyl)-1H-indoles (Target Compound)

To a solution of the above crude intermediates **2a**–**2p** (1 eq.), 5-iodo-1,2,3-trimethoxybenzene (0.8 eq.), CuI (0.15 eq.), and Cs_2_CO_3_ (0.6 eq.) in DMF (6 mL) were mixed in a round-bottom flask under argon. The mixture was stirred at 150 °C for 16 h, and the reaction was monitored by TLC. The reaction was quenched by water and extracted with ethyl acetate (3 × 20 mL). The combined organic layers were washed with brine, dried over Na_2_SO_4_, filtered, and concentrated in vacuo. Purification by flash chromatography yielded the target compounds **3a**–**3p**.

*6-phenyl-1-(3,4,5-trimethoxyphenyl)-1H-indole* (**3a**)

Pink solid; yield: 57.3%. m.p. 127.1–127.7 °C. ^1^H NMR (500 MHz, CDCl_3_) δ 7.75 (d, *J* = 8.0 Hz, 2H), 7.64 (dd, *J* = 8.0, 1.4 Hz, 2H), 7.46–7.43 (m, 3H), 7.34 (d, *J* = 3.0 Hz, 2H), 6.76 (s, 2H), 6.70 (d, *J* = 3.0 Hz, 1H), 3.94 (s, 3H), 3.90 (s, 6H). ^13^C NMR (126 MHz, CDCl_3_) δ 154.01, 142.41, 137.03, 136.86, 136.14, 135.63, 128.98, 128.85, 128.52, 127.51, 126.85, 121.49, 120.40, 109.12, 103.27, 102.66, 61.18, 56.49. HRMS (ESI): calcd. for C_23_H_21_NO_3_, [M + H]^+^ 360.1594; found: 360.1603.

*3-(1-(3,4,5-trimethoxyphenyl)-1H-indol-6-yl)benzonitrile* (**3b**)

White solid; yield: 35.7%. m.p. 134.6–135.0 °C. ^1^H NMR (500 MHz, CDCl_3_) δ 7.88 (d, *J* = 1.5 Hz, 1H), 7.84 (dt, *J* = 7.5, 1.5 Hz, 1H), 7.78 (d, *J* = 8.0 Hz, 1H), 7.69–7.65 (m, 2H), 7.59 (dt, *J* = 7.5, 1.5 Hz, 1H), 7.52 (t, *J* = 7.5 Hz, 1H), 7.40–7.36 (m, 2H), 6.73 (s, 2H), 6.71 (dd, *J* = 3.5, 1.0 Hz, 1H), 3.95 (s, 3H), 3.90 (s, 6H). ^13^C NMR (126 MHz, CDCl_3_) δ 154.10, 143.65, 137.34, 136.87, 135.28, 133.57, 131.79, 131.00, 130.19, 129.76, 129.66, 129.22, 121.95, 119.93, 119.14 (C≡N), 112.99, 109.18, 103.30, 102.82, 61.19, 56.52. HRMS (ESI): calcd. for C_24_H_20_N_2_O_3_, [M + H]^+^ 385.1547; found: 385.1550.

*6-(m-tolyl)-1-(3,4,5-trimethoxyphenyl)-1H-indole* (**3c**)

White solid; yield: 84.2%. m.p. 131.0–131.4 °C. ^1^H NMR (500 MHz, CDCl_3_) δ 7.74 (d, *J* = 8.5 Hz, 2H), 7.45–7.42 (m, 3H), 7.37–7.30 (m, 2H), 7.15 (d, *J* = 7.5 Hz, 1H), 6.76 (s, 2H), 6.70 (d, *J* = 3.5 Hz, 1H), 3.95 (s, 3H), 3.90 (s, 6H), 2.43 (s, 3H). ^13^C NMR (126 MHz, CDCl_3_) δ 154.01, 142.41, 138.41, 136.99, 136.83, 136.29, 135.68, 128.91, 128.76, 128.47, 128.35, 127.62, 124.62, 121.42, 120.47, 109.10, 103.27, 102.64, 61.19, 56.48, 21.72. HRMS (ESI): calcd. for C_24_H_23_NO_3_, [M + H]^+^ 374.1751; found: 374.1764.

*6-(2-ethylphenyl)-1-(3,4,5-trimethoxyphenyl)-1H-indole* (**3d**)

Yellow solid; yield: 75.6%. m.p. 87.3–87.8 °C. ^1^H NMR (500 MHz, CDCl_3_) δ 7.71 (d, *J* = 8.1 Hz, 1H), 7.48 (d, *J* = 0.6 Hz, 1H), 7.35 (d, *J* = 3.2 Hz, 1H), 7.31 (dd, *J* = 6.0, 1.6 Hz, 2H), 7.25–7.21 (m, 2H), 7.15 (dd, *J* = 8.1, 1.6 Hz, 1H), 6.74–6.71 (m, 3H), 3.90 (s, 3H), 3.88 (s, 6H), 2.66 (q, *J* = 7.5 Hz, 2H), 1.11 (t, *J* = 7.5 Hz, 3H). ^13^C NMR (126 MHz, CDCl_3_) δ 153.94, 142.63, 141.96, 136.97, 136.62, 136.22, 135.65, 130.49, 128.60, 127.90, 127.35, 125.57, 122.41, 120.61, 111.16, 103.27, 102.61, 61.15, 56.41, 26.40, 15.89. HRMS (ESI): calcd. for C_25_H_25_NO_3_, [M + H]^+^ 388.1907; found: 388.1913.

*6-(4-ethylphenyl)-1-(3,4,5-trimethoxyphenyl)-1H-indole* (**3e**)

Pink solid; yield: 22.9%. m.p. 100.1–100.5 °C. ^1^H NMR (500 MHz, CDCl_3_) δ 7.75–7.73 (m, 2H), 7.56 (d, *J* = 8.5 Hz, 2H), 7.44 (dd, *J* = 8.0, 1.4 Hz, 1H), 7.33 (d, *J* = 3.0 Hz, 1H), 7.28 (d, *J* = 8.5 Hz, 2H), 6.76 (s, 2H), 6.69 (dd, *J* = 3.0, 0.5 Hz, 1H), 3.94 (s, 3H), 3.90 (s, 6H), 2.71 (q, *J* = 7.6 Hz, 2H), 1.29 (t, *J* = 7.6 Hz, 3H). ^13^C NMR (126 MHz, CDCl_3_) δ 154.00, 142.96, 139.79, 136.96, 136.87, 136.14, 135.68, 128.82, 128.39, 128.32, 127.42, 121.41, 120.35, 108.91, 103.27, 102.64, 61.18, 56.49, 28.62, 15.75. HRMS (ESI): calcd. for C_25_H_25_NO_3_, [M + H]^+^ 388.1907; found: 388.1915.

*6-(2-isopropylphenyl)-1-(3,4,5-trimethoxyphenyl)-1H-indole* (**3f**)

Yellow solid; yield: 65.4%. m.p. 112.9–112.5 °C. ^1^H NMR (500 MHz, CDCl_3_) δ 7.71 (d, *J* = 7.5 Hz, 1H), 7.49–7.44 (m, 1H), 7.40 (d, *J* = 7.5 Hz, 1H), 7.38–7.31 (m, 2H), 7.25–7.18 (m, 2H), 7.13 (d, *J* = 8.0 Hz, 1H), 6.74 (s, 2H), 6.72 (d, *J* = 3.0 Hz, 1H), 3.90 (s, 3H), 3.87 (s, 6H), 3.16 (*p*, *J* = 7.0 Hz, 1H), 1.17 (d, *J* = 6.8 Hz, 6H). ^13^C NMR (126 MHz, CDCl_3_) δ 153.93, 146.73, 142.09, 136.94, 136.77, 136.19, 135.64, 130.41, 128.58, 127.86, 127.57, 125.55, 125.28, 122.48, 120.53, 111.19, 103.29, 102.58, 61.15, 56.39, 29.57, 24.46. HRMS (ESI): calcd. for C_26_H_27_NO_3_, [M + H]^+^ 402.2064; found: 402.2069.

*2-methyl-5-(1-(3,4,5-trimethoxyphenyl)-1H-indol-6-yl)benzonitrile* (**3g**)

Yellow solid; yield: 71.6%. m.p. 157.4–158.0 °C. ^1^H NMR (500 MHz, CDCl_3_) δ 7.83 (d, *J* = 2.0 Hz, 1H), 7.76 (d, *J* = 8.0 Hz, 1H), 7.72 (dd, *J* = 8.0, 2.0 Hz, 1H), 7.66–7.64 (m, 1H), 7.40–7.33 (m, 3H), 6.73 (s, 2H), 6.70 (dd, *J* = 3.5, 1.0 Hz, 1H), 3.95 (s, 3H), 3.90 (s, 6H), 2.58 (s, 3H). ^13^C NMR (126 MHz, CDCl_3_) δ 154.09, 140.77, 140.11, 137.27, 136.85, 135.34, 133.59, 131.62, 131.19, 130.81, 129.57, 129.01, 121.86, 119.83 (C≡N), 113.33, 108.93, 103.30, 102.76, 61.19, 56.51, 20.18. HRMS (ESI): calcd. for C_25_H_22_N_2_O_3_, [M + H]^+^ 399.1703; found: 399.1720.

*6-(3-(trifluoromethoxy)phenyl)-1-(3,4,5-trimethoxyphenyl)-1H-indole* (**3h**)

Yellow solid; yield:28.3%. m.p. 81.7–82.3 °C. ^1^H NMR (500 MHz, CDCl_3_) δ 7.76 (d, *J* = 8.0 Hz, 1H), 7.73–7.71 (m, 1H), 7.55 (dt, *J* = 8.0, 1.5 Hz, 1H), 7.48–7.46 (m, 1H), 7.44 (t, *J* = 8.0 Hz, 1H), 7.41 (dd, *J* = 8.5, 1.5 Hz, 1H), 7.37 (d, *J* = 3.0 Hz, 1H), 7.19–7.15 (m, 1H), 6.75 (s, 2H), 6.71 (dd, *J* = 3.0, 0.5 Hz, 1H), 3.94 (s, 3H), 3.94 (s, 3H), 3.90 (s, 6H). ^13^C NMR (126 MHz, CDCl_3_) δ 154.08, 149.82 (d, *J* = 1.7 Hz), 144.55, 137.12, 136.76, 135.45, 134.48, 130.13, 129.42, 129.05, 125.79, 121.75, 120.16, 119.98, 119.67, 119.07, 109.23, 103.35, 102.57, 61.20, 56.48. HRMS (ESI): calcd. for C_24_H_20_F_3_NO_4_, [M + H]^+^ 444.1417; found: 444.1444.

*6-(4-butylphenyl)-1-(3,4,5-trimethoxyphenyl)-1H-indole* (**3i**)

Yellow solid; yield: 37.3%. m.p. 77.1–77.8 °C. ^1^H NMR (500 MHz, CDCl_3_) δ 7.74–7.72 (m, 2H), 7.54 (d, *J* = 8.0 Hz, 2H), 7.44 (dd, *J* = 8.5, 1.5 Hz, 1H), 7.33 (d, *J* = 3.2 Hz, 1H), 7.24 (s, 1H), 6.75 (s, 2H), 6.69 (d, *J* = 3.2 Hz, 1H), 3.94 (s, 3H), 3.90 (s, 6H), 2.66 (t, *J* = 7.5 Hz, 2H), 1.65 (*p*, *J* = 7.5 Hz, 2H), 1.42–1.36 (m, 2H), 0.96 (t, *J* = 7.4 Hz, 3H). ^13^C NMR (126 MHz, CDCl_3_) δ 153.98, 141.63, 139.71, 136.94, 136.86, 136.15, 135.68, 129.93, 128.81, 128.30, 127.32, 121.40, 120.34, 108.89, 103.26, 102.61, 61.18, 56.48, 35.40, 33.82, 22.55, 14.11. HRMS (ESI): calcd. for C_27_H_29_NO_3_, [M + H]^+^ 416.2220; found: 416.2248.

*6-(4-(methylthio)phenyl)-1-(3,4,5-trimethoxyphenyl)-1H-indole* (**3j**)

Pink solid; yield: 57.8%. m.p. 110.7–111.4 °C. ^1^H NMR (500 MHz, CDCl_3_) δ 7.75–7.70 (m, 2H), 7.57–7.54 (m, 2H), 7.42 (dd, *J* = 8.2, 2.0 Hz, 1H), 7.35–7.33 (m, 2H), 7.32 (d, *J* = 2.0 Hz, 1H), 6.75 (s, 2H), 6.69 (dd, *J* = 3.2, 0.8 Hz, 1H), 3.94 (s, 3H), 3.90 (s, 6H), 2.52 (s, 3H). ^13^C NMR (126 MHz, CDCl_3_) δ 154.02, 139.31, 137.03, 136.96, 136.85, 135.59, 135.42, 129.00, 128.50, 127.82, 127.23, 121.55, 120.0, 108.77, 103.30, 102.62, 61.17, 56.49, 16.17. HRMS (ESI): calcd. for C_24_H_23_NO_3_S, [M + H]^+^ 406.1471; found: 406.1498.

*6-(4-isopropylphenyl)-1-(3,4,5-trimethoxyphenyl)-1H-indole* (**3k**)

White solid; yield: 24.3%. m.p. 115.8–116.3 °C. ^1^H NMR (500 MHz, CDCl_3_) δ 7.75–7.72 (m, 2H), 7.57 (d, *J* = 8.0 Hz, 2H), 7.44 (dd, *J* = 8.5, 1.5 Hz, 1H), 7.32 (d, *J* = 3.2 Hz, 1H), 7.29 (d, *J* = 8.2 Hz, 2H), 6.76 (s, 2H), 6.69 (d, *J* = 3.2 Hz, 1H), 3.94 (s, 3H), 3.90 (s, 6H), 2.96 (*p*, *J* = 6.9 Hz, 1H), 1.30 (d, *J* = 6.9 Hz, 6H). ^13^C NMR (126 MHz, CDCl_3_) δ 154.00, 147.57, 139.93, 136.99, 136.88, 136.15, 135.68, 128.81, 128.32, 127.42, 126.95, 121.40, 120.38, 108.94, 103.27, 102.65, 61.18, 56.50, 33.90, 24.17. HRMS (ESI): calcd. for C_26_H_27_NO_3_, [M + H]^+^ 402.2064; found: 402.2073.

*2-(1-(3,4,5-trimethoxyphenyl)-1H-indol-6-yl)benzonitrile* (**3l**)

White solid; yield: 54.8%. m.p. 163.2–163.8 °C. ^1^H NMR (500 MHz, CDCl_3_) δ 7.82–7.78 (m, 1H), 7.79 (dd, *J* = 8.0, 1.0 Hz, 1H), 7.75 (ddd, *J* = 7.8, 1.4, 0.6 Hz, 1H), 7.62 (td, *J* = 7.7, 1.4 Hz, 1H), 7.56 (ddd, *J* = 7.9, 1.4, 0.6 Hz, 1H), 7.43–7.36 (m, 2H), 7.30 (dd, *J* = 8.0, 1.5 Hz, 1H), 6.80 (s, 2H), 6.73 (dd, *J* = 3.0, 1.0 Hz, 1H), 3.93 (s, 6H), 3.92 (s, 3H). ^13^C NMR (126 MHz, CDCl_3_) δ 154.09, 146.79, 137.03, 136.01, 135.35, 133.79, 132.78, 132.53, 130.51, 129.64, 129.42, 127.08, 121.63, 121.38, 119.40 (C≡N), 111.57, 111.48, 103.36, 102.49, 61.12, 56.62. HRMS (ESI): calcd. for C_24_H_20_N_2_O_3_, [M + H]^+^ 385.1547; found: 385.1564.

*4-(1-(3,4,5-trimethoxyphenyl)-1H-indol-6-yl)benzonitrile* (**3m**)

Pink solid; yield: 50.2%. m.p. 137.8–138.6 °C. ^1^H NMR (500 MHz, CDCl_3_) δ 7.78 (d, *J* = 8.0 Hz, 1H), 7.72 (d, *J* = 1.5 Hz, 1H), 7.71 (s, 4H), 7.42 (dd, *J* = 8.2, 1.6 Hz, 1H), 7.38 (d, *J* = 3.2 Hz, 1H), 6.73 (s, 2H), 6.71 (dd, *J* = 3.0, 1.0 Hz, 1H), 3.94 (s, 3H), 3.90 (s, 6H). ^13^C NMR (126 MHz, CDCl_3_) δ 154.10, 146.89, 137.32, 136.84, 135.26, 133.80, 132.68, 129.92, 129.4, 127.93, 121.93, 120.02, 119.24 (C≡N), 110.28, 109.38, 103.36, 102.78, 61.18, 56.53. HRMS (ESI): calcd. for C_24_H_20_N_2_O_3_, [M + H]^+^ 385.1547; found: 385.1547.

*3-fluoro-4-(1-(3,4,5-trimethoxyphenyl)-1H-indol-6-yl)benzonitrile* (**3n**)

White solid; yield: 15.6%. m.p. 164.7–165.4 °C. ^1^H NMR (500 MHz, CDCl_3_) δ 7.79–7.77 (m, 2H), 7.61 (t, *J* = 7.5 Hz, 1H), 7.51 (dd, *J* = 8.0, 2.0 Hz, 1H), 7.45 (dd, *J* = 10.0, 1.5 Hz, 1H), 7.41 (d, *J* = 3.0 Hz, 1H), 7.34 (dt, *J* = 8.0, 1.5 Hz, 1H), 6.74 (s, 2H), 6.72 (d, *J* = 3.0 Hz, 1H), 3.92 (s, 3H), 3.90 (s, 6H). ^13^C NMR (126 MHz, CDCl_3_) δ 160.36, 158.37, 154.08, 137.15, 136.18, 135.53, 135.43, 135.19, 132.0, 131.99, 129.94, 129.67, 128.47, 128.44, 127.87, 121.64, 121.30, 120.16, 119.94, 117.91, 111.68, 111.64, 111.60, 111.53, 103.46, 102.43, 61.17, 56.46. HRMS (ESI): calcd. for C_24_H_19_FN_2_O_3_, [M + H]^+^ 403.1452; found: 403.1467.

*6-(4-Phenoxyphenyl)-1-(3,4,5-trimethoxyphenyl)-1H-indole* (**3o**)

White solid; yield: 18.6%. m.p. 140.2–140.9 °C. ^1^H NMR (500 MHz, CDCl_3_) δ 7.74 (dd, *J* = 8.0, 1.0 Hz, 1H), 7.71 (*p*, *J* = 1.0 Hz, 1H), 7.59 (d, *J* = 8.5 Hz, 2H), 7.42 (dd, *J* = 8.0, 1.5 Hz, 1H), 7.38–7.32 (m, 3H), 7.14–7.05 (m, 5H), 6.75 (s, 2H), 6.69 (dd, *J* = 3.0, 1.0 Hz, 1H), 3.94 (s, 3H), 3.90 (s, 6H). ^13^C NMR (126 MHz, CDCl_3_) δ 157.39, 156.53, 154.02, 137.53, 137.06, 136.83, 135.62, 135.50, 129.90, 128.95, 128.72, 128.35, 123.42, 121.52, 120.23, 119.23, 119.06, 108.83, 103.28, 102.69, 61.18, 56.51. HRMS (ESI): calcd. for C_29_H_25_NO_4_, [M + H]^+^ 452.1856; found: 452.1876.

*2-(1-(3,4,5-trimethoxyphenyl)-1H-indol-6-yl)benzo[d]thiazole* (**3p**)

White solid; yield: 16.6%. m.p. 162.3–162.7 °C. ^1^H NMR (500 MHz, CDCl_3_) δ 8.34–8.30 (m, 1H), 8.07–8.01 (m, 1H), 7.91 (dd, *J* = 8.5, 1.5 Hz, 1H), 7.89–7.87 (m, 1H), 7.77 (dd, *J* = 8.0, 0.5 Hz, 1H), 7.47 (ddd, *J* = 8.2, 7.2, 1.2 Hz, 1H), 7.43 (d, *J* = 3.0 Hz, 1H), 7.35 (ddd, *J* = 8.2, 7.2, 1.1 Hz, 1H), 6.76 (s, 2H), 6.72 (dd, *J* = 3.2 Hz, 0.8 Hz, 1H), 3.97 (s, 3H), 3.93 (s, 6H). ^13^C NMR (126 MHz, CDCl_3_) δ 169.37, 154.32, 154.03, 137.24, 136.29, 135.04, 134.97, 131.41, 130.66, 128.09, 126.23, 124.82, 122.87, 121.61, 121.51, 120.22, 110.08, 103.63, 102.60, 61.09, 56.43. HRMS (ESI): calcd. for C_24_H_20_N_2_O_3_S, [M + H]^+^ 417.1267; found: 417.1295.

### 3.5. Biological Assays

#### 3.5.1. BLI Analysis

BLI assays were performed on an Octet RED96 instrument (FortéBio, Pall Life Sciences, Menlo Park, CA, USA) at 25 °C in PBS buffer supplemented with 0.02% (*v*/*v*) Tween-20 as the running buffer. Biotinylated tubulin proteins (3 μg/mL) were immobilized onto Super Streptavidin (SSA) biosensors. Association and dissociation were monitored for 60 s each. Compounds were tested at serial concentrations of 200, 100, 50, 25, 12.5, and 6.25 μM. Reference subtraction was performed using sensors incubated in running buffer without protein loading to correct for baseline drift. Data were analyzed using Octet Data Analysis software (version 9.0), and equilibrium dissociation constants (K_D_) were determined by fitting the curves to a 1:1 binding model. All measurements were performed in triplicate, and results are reported as mean ± SD.

#### 3.5.2. Cell Lines and Cell Culture

Human breast cancer cells (MCF-7), human lung cancer cells (A549), human cervical cancer cells (HeLa), human triple-negative breast cancer cells (MDA-MB-231), and human malignant melanoma cells (A375) were cultured in DMEM. Mouse melanoma cells (B16F10) were maintained in RPMI-1640 medium. All media were supplemented with 10% fetal bovine serum and 1% penicillin-streptomycin (Life Technologies, Carlsbad, CA, USA) and maintained at 37 °C in a humidified atmosphere with 5% CO_2_.

#### 3.5.3. Cytotoxicity Assay

The cytotoxicity of the test compounds was evaluated against MCF-7, A549, HeLa, MDA-MB-231, A375, and B16F10 using the CCK-8 assay. Cells were seeded into 96-well plates at a density of 5000 cells/well. After removing the medium, 100 μL of medium with 0.1% DMSO containing test compounds at different concentrations was added to each well and incubated at 37 °C for another 48 h. The CCK-8 solution was added and incubated for another 1 h, and then, the absorbance was detected with a microplate reader at a wavelength of 450 nm. The IC_50_ values were calculated by nonlinear regression analysis using GraphPad Prism 8.0.2. All the experiments were repeated at least three times.

#### 3.5.4. In Vitro Antiproliferative Assay

Cells were seeded into 96-well plates at a density of 5000 cells/well. After being treated for 48 h with compound **3g** at the indicated concentrations or time points, the CCK-8 was added and incubated for another 1 h. Cell viability was detected with a microplate reader at a wavelength of 450 nm.

#### 3.5.5. Colony Formation Assay

MCF-7 cells (1000 cells/well) were counted and seeded in 6-well plates. After being cultured for 24 h at 37 °C, the medium was replaced with medium added with compound **3g** at the indicated concentration. After 24 h of treatment, the medium was changed to normal. After being cultured for another 10–14 days, the colonies were fixed with 4% paraformaldehyde for 30 min and stained with 0.1% crystal violet for 15 min. Following washing with PBS, the colonies were then photographed and quantified using Image J 1.54g.

#### 3.5.6. In Vitro Tubulin Polymerization Assay

Porcine brain tubulin protein was dissolved in EM buffer containing 5% glycerol (80 mM PIPES, 2 mM GTP, 2 mM MgCl_2_, and 0.5 mM EGTA, pH 6.9). Compounds were diluted in EM buffer to final concentrations of 50, 25, 10, 5, and 2.5 μM and added to a pre-warmed 384-well plate, followed by pre-incubation at 37 °C. Subsequently, 14 μL of the tubulin-GTP mixture was added to each well containing the test compounds. Tubulin polymerization was monitored by measuring absorbance at 340 nm every minute for 60 min. Colchicine and paclitaxel were used as positive controls, and 0.1% DMSO served as the negative control. Each assay was performed in triplicate.

#### 3.5.7. Wound Healing Assay

MCF-7 cells were seeded and incubated in a 6-well plate overnight. Scratches were made in confluent monolayers with a 200 μL pipette tip. Then, wounds were washed twice with media to remove debris and uprooted cells. Cells were treated with various concentrations (0, 0.1, 1, and 10 μM) of compound **3g**. Images were obtained using phase contrast microscopy at 0 and 24 h. The migration distance of cells in the wound area was quantified using ImageJ.

#### 3.5.8. Transwell Assay

MCF-7 cells were seeded into six-well culture plates at a density of 5 × 10^5^ cells per well for 24 h. After treatment with compounds **3g** at different concentrations for 24 h, cells were harvested and inoculated on transwell filters (8 μM pore size, Millipore, Billerica, MA, USA) in 24-well culture plates. The cells in the top chamber were cultured in the FBS-free medium, and those in the bottom chamber were cultured in the medium containing 10% FBS. The plates were cultivated for an additional 48 h, and the cells that had migrated to the bottom of the top chambers were fixed with 4% paraformaldehyde and then stained with 0.1% crystal violet. Images of migrated cells were obtained using an inverted microscope (Olympus IX73, Olympus Corporation, Tokyo, Japan).

#### 3.5.9. Immunofluorescence Staining

MCF-7 cells were seeded on glass coverslips in 24-well plates and then treated with vehicle control 0.1% DMSO, **3g** (0.1, 1, 10 μM) for 24 h. The cells were fixed with 4% paraformaldehyde and then permeabilized with PBS containing 0.2% Triton X-100. After blocking for 30 min by adding 100 μL of goat serum albumin at room temperature, cells were incubated with a *β*-tubulin polyclonal antibody (Proteintech, Wuhan Sanying Biotechnology, Wuhan, China, Cat# 10094-1-AP, 1:500 dilution) at 37 °C for 12 h. Then, the cells were washed three times with PBS and incubated with a secondary antibody (Beyotime, Shanghai, China, Cat# A0516, 1:500 dilution) for 1 h at room temperature. Nuclei were labeled with 4,6-diamidino-2-phenylindole (DAPI). Cells were finally visualized using a fluorescence microscope (Nikon TI2-E+A1R, Nikon Corporation, Tokyo, Japan).

#### 3.5.10. Cell Cycle Analysis

MCF-7 cells were seeded in 6-well plates and cultured overnight. Cells were then treated with compound **3g** at concentrations of 0.1, 1, and 10 μM for 48 h. After treatment, cells were collected and fixed in 70% ethanol at 4 °C overnight. The fixed cells were washed and resuspended in staining buffer containing RNase A and propidium iodide (PI), followed by incubation in the dark at 37 °C for 30 min. DNA content was subsequently analyzed by flow cytometry.

#### 3.5.11. Cancer Cell Apoptosis Analysis

MCF-7 cells were seeded into 6-well plates and cultured overnight. After treating with compound **3g** (0.1, 1, and 10 μM) for 48 h, cells were harvested and suspended in binding buffer containing annexin V-FITC (0.5 mg/mL) and PI (0.5 mg/mL). After that, samples were incubated for 20 min in the dark and analyzed with a flow cytometer.

#### 3.5.12. In Vivo Antitumor Evaluation

All animal procedures were approved by the Animal Ethics Committee of the Naval Medical University (permit number: EC11-055). Male BALB/c mice (6–8 weeks old, average initial body weight approximately 18–20 g) were purchased from Shanghai SLAC Laboratory Animal Co., Ltd. The inhibitory effect of compound **3g** was evaluated using a subcutaneous B16-F10 melanoma xenograft model. Log-phase B16F10 cells were suspended in PBS and subcutaneously injected into mice (200 μL containing 8 × 10^5^ cells per mouse) to establish tumors. Mice were randomly divided into control and treatment groups (n = 8). Compound **3g** and paclitaxel were dissolved in a vehicle consisting of DMSO:PEG:Tween-80:saline (*v*/*v*/*v*/*v*, 5:35:10:50) to the desired concentrations. Treatments (paclitaxel, 10 mg/kg; compound **3g**, 20 and 50 mg/kg, doses calculated on a body-weight basis) and vehicle were administered once daily via intraperitoneal injection for 14 consecutive days. Body weights were monitored throughout the study to assess acute toxicity. Tumor volumes were measured using calipers and calculated with the formula: *a × b^2^ × 0.5*, where *a* and *b* represent the larger and smaller tumor diameters, respectively. On day 14, mice were sacrificed and tumors were excised and weighed. TGI was calculated using the formula: TGI (%) = [1 − *W_t_/W_v_*] × 100%, where *W_t_* and *W_v_* are the average tumor weights of the treatment and vehicle groups, respectively. Major organs (liver and kidneys) were collected, fixed in 4% paraformaldehyde, processed into paraffin sections, stained with hematoxylin and eosin (H&E), and examined microscopically.

## 4. Conclusions

Using a virtual FragLites screening workflow combined with an FBDD strategy, we successfully designed and synthesized a series of 6-substituted-1-(3,4,5-trimethoxyphenyl)-*1H*-indole derivatives as potential tubulin inhibitors. BLI assays revealed that most compounds exhibited measurable binding to tubulin, with compounds **3g**, **3c**, **3d**, **3l**, and **3b** showing the strongest affinities (K_D_ values in the low micromolar range). The enhanced binding of **3g** can be attributed to the 3-cyano-4-methyl substitution at the indole 6-position, which likely strengthens hydrophobic and halogen bonding interactions within the colchicine-binding site. The correlation between K_D_ values and IC_50_ in cell viability assays indicates that cellular antiproliferative effects are largely mediated through tubulin engagement.

Compound **3g** demonstrated the most potent antiproliferative activity among the tested derivatives, with low micromolar IC_50_ values across multiple cancer cell lines, including MCF-7, MDA-MB-231, A549, HeLa, A375, and B16-F10. Mechanistic studies showed that **3g** disrupted tubulin polymerization in vitro in a concentration-dependent manner, resulting in G_2_/M cell-cycle arrest and apoptosis in MCF-7 cells. These cellular responses align with the established mechanism of microtubule-destabilizing agents, such as colchicine and combretastatin A-4, which perturb spindle formation, activate the mitotic checkpoint, and trigger apoptotic cell death [32,33].

In vivo evaluation in the B16-F10 melanoma xenograft model demonstrated that **3g** produced dose-dependent tumor growth inhibition (TGI = 23.34% and 44.18% at 20 and 50 mg/kg, respectively). Although slightly less efficacious than paclitaxel, which stabilizes microtubules via a different mechanism, the antitumor activity of **3g** is comparable to that of other colchicine-binding site inhibitors reported in the literature [34]. Importantly, histopathological examination of major organs revealed no overt toxicity at the tested doses, suggesting favorable systemic tolerability. However, more comprehensive pharmacokinetic and toxicological studies, including biochemical enzyme assays, are warranted to fully evaluate the safety profile.

Taken together, our results indicate that the 6-substituted-1-(3,4,5-trimethoxyphenyl)-*1H*-indole scaffold, particularly compound **3g**, is a promising lead for the development of new tubulin polymerization inhibitors. The observed correlation between structural modification, binding affinity, and cellular activity provides guidance for further optimization. Future work will focus on improving pharmacokinetic properties, expanding the structure–activity relationship, and performing detailed preclinical toxicity evaluations to further characterize compound **3g**.

## Figures and Tables

**Table 1:** Binding Affinities of Compounds to Tubulin Protein (K_D_ Values [μM] Were Determined from the Biolayer Interferometry Assay).

ID	K_D_ (μM)	ID	K_D_ (μM)
**3a**	39 ± 2.60	**3i**	NB
**3b**	29 ± 4.10	**3j**	NB
**3c**	23 ± 2.12	**3k**	ND
**3d**	24 ± 2.13	**3l**	26 ± 3.38
**3e**	110 ± 14.46	**3m**	NB
**3f**	47 ± 5.62	**3n**	220 ± 30.80
**3g**	13 ± 1.17	**3o**	31 ± 3.41
**3h**	ND	**3p**	NB
Colchicine	8.60 ± 1.20		

ND = not detected (no specific binding detected); NB = no binding or very weak binding.

**Table 2:** In Vitro Antiproliferative Activities of Compounds against Six Cancer Cell Lines.

Compd.	IC_50_ (μM)					
	MCF-7	MDA-MB-231	A549	Hela	A375	B16-F10
**3a**	10.13 ± 0.68	78.92 ± 0.57	36.60 ± 1.78	>100	1.16 ± 0.12	45.36 ± 1.11
**3b**	3.33 ± 0.28	42.93 ± 3.13	41.20 ± 3.42	38.52 ± 2.25	27.53 ± 2.50	3.78 ± 0.14
**3c**	9.15 ± 0.21	37.72 ± 0.89	92.44 ± 8.58	66.50 ± 6.21	53.16 ± 4.06	59.21 ± 5.86
**3d**	34.22 ± 1.62	23.37 ± 0.44	60.24 ± 4.17	46.60 ± 3.65	24.84 ± 0.16	6.49 ± 0.40
**3e**	>100	>100	>100	>100	>100	>100
**3f**	17.39 ± 0.35	21.37 ± 0.12	87.43 ± 3.45	41.80 ± 1.11	21.07 ± 1.17	6.67 ± 0.29
**3g**	2.94 ± 0.56	1.61 ± 0.004	6.30 ± 0.30	6.10 ± 0.31	0.57 ± 0.01	1.69 ± 0.41
**3h**	>100	>100	>100	>100	>100	>100
**3i**	>100	>100	>100	>100	>100	>100
**3j**	>100	>100	>100	>100	>100	>100
**3k**	>100	>100	>100	>100	>100	>100
**3l**	>100	>100	>100	>100	>100	>100
**3m**	>100	>100	>100	>100	>100	>100
**3n**	14.17 ± 0.51	41.37 ± 1.21	48.01 ± 2.56	65.00 ± 3.20	16.10 ± 0.38	68.94 ± 3.17
**3o**	>100	>100	>100	>100	>100	>100
**3p**	>100	>100	>100	>100	>100	>100
Colchicine	0.26 ± 0. 08	0.48 ± 0.03	0.082 ± 0.006	0.027 ± 0.002	0.110 ± 0.02	0.013 ± 0.001

IC_50_: Values [μM] Were Determined from the Cell Viability Assay for 48 h.

## Data Availability

Data are contained within the article and Supplementary Materials.

## Supplementary Materials

The following supporting information can be downloaded at https://www.mdpi.com/article/10.3390/molecules30234538/s1. Figures S1–S16: NMR, HRMS, HPLC Spectra of All Products; Figures S17–S33: Analysis of the affinity of **3a–3g** and colchicine to tubulin by bio-layer interferometry (BLl); Figure S34: Superposition of the docking poses of **3g** (sea green) and the original hit compound (pink) in the colchicine-binding site of tubulin; Figure S35: Predicted binding pose of compound **3g** in the colchicine-binding site of tubulin.
