# Supplementary material for: Design, Synthesis and Anticancer Activity of 6-Substituted-1-(3,4,5-trimethoxyphenyl)-1H-indole Against Tubulin Polymerisation
Source: Molecules. 2025 Nov 24;30(23):4538. doi: 10.3390/molecules30234538 (PMC12693167; doi:10.3390/molecules30234538)
Supplement: Supplementary file 1 [file molecules-30-04538-s001.zip › molecules-3967069-supplementary.pdf]

Supplementary Information

Design, Synthesis and Anticancer Activity of 6-Substituted-1-(3,4,5-trimethoxyphenyl)-*1H*-indole Against Tubulin Polymerisation

Yuanna Gu [1,†], Conghao Gai [2,†], Sijie Zou [3], Yan Song [3], Juan Zhang [3], Qingjie Zhao [2,*], Xiaoyun Chai [2,*] and Peipei Wang [1,4,*]

[1] College of Food Science and Technology, Shanghai Ocean University, Shanghai 201306, China; giwianna0604@163.com
[2] Department of Organic Chemistry, School of Pharmacy, Naval Medical University, Shanghai 200433, China; c.gai2@outlook.com
[3] Department of Pharmacy, PLA Naval Medical Center, Naval Medical University, Shanghai 200052, China; 18755222267@163.com (S.Z.); songyan455@126.com (Y.S.); lyzj0706@163.com (J.Z.)
[4] Marine Biomedical Science and Technology Innovation Platform of Lin-Gang Special Area, Shanghai 201306, China
* Correspondence: qjzhao_325@126.com (Q.Z.); chaixy1207@163.com (X.C.); ppwang@shou.edu.cn (P.W.)
† These authors contributed equally to this work.

Content

Analytical Equipment .... - 2 -

NMR, HRMS, HPLC spectra .... - 3 -

Analysis of the affinity of 3a-3g to tubulin by bio-layer interferometry (BLI) .... - 26 -

Docking Studies of the Compound 3g .... - 35 -

Analytical Equipment

Melting points were measured using a Stuart automatic melting point SMP40 apparatus or a Shanghai ShenGuang WRR apparatus. All solvents were used after appropriate distillation or purification. ^{1}H NMR and ^{13}C NMR spectra were recorded with Bruker Advance 300/400/500 MHz spectrometer (Bruker Company, Germany) in the indicated sol vents ($CDCl_3$ or DMSO-d_6, tetramethylsilane (TMS) as internal stan dard): the values of the chemical shifts are expressed in δ values (ppm) and the coupling constants (*J*) in hertz. High-resolution mass spectra (HRMS) were measured with an Aglilent Technologies 6538 UHD Accurate-Mass Q-TOF MS spectrometer using ESI.

LC-MS analyses were conducted using a Waters Acquity UPLC system with photo diode array (PDA) and evaporating light scattering detector (ELSD) or using the ESI mass spectra which were performed by Zichao Ding on an Agilent Technologies 6120 Quadrupole LC-MS. When a 2 min gradient was used, the sample was eluted on an Acquity UPLC BEH C18, 1.7μm, 2.1 x 50mm, with a flow rate of 1 ml/min using 10-100% 0.1% trifluoroacetic acid in MeCN. Analytical purity of compounds was determined using Waters XTerra RP18, 5 μm (4.6 × 150 mm) column at 1 ml/min using either 0.1% aq. and MeCN or 0.1% aq. trifluoroacetic acid and MeCN with a gradient of 10-100% over 20 min. When a 12 min gradient was used, the sample was eluted on ZORBAX Eclipse XDB-C18, 3.5 μm, 4.6 x 100 mm, with a flow rate of 1 ml/min using 30-70% 0.1% trifluoroacetic acid in MeCN.

NMR, HRMS, HPLC spectra

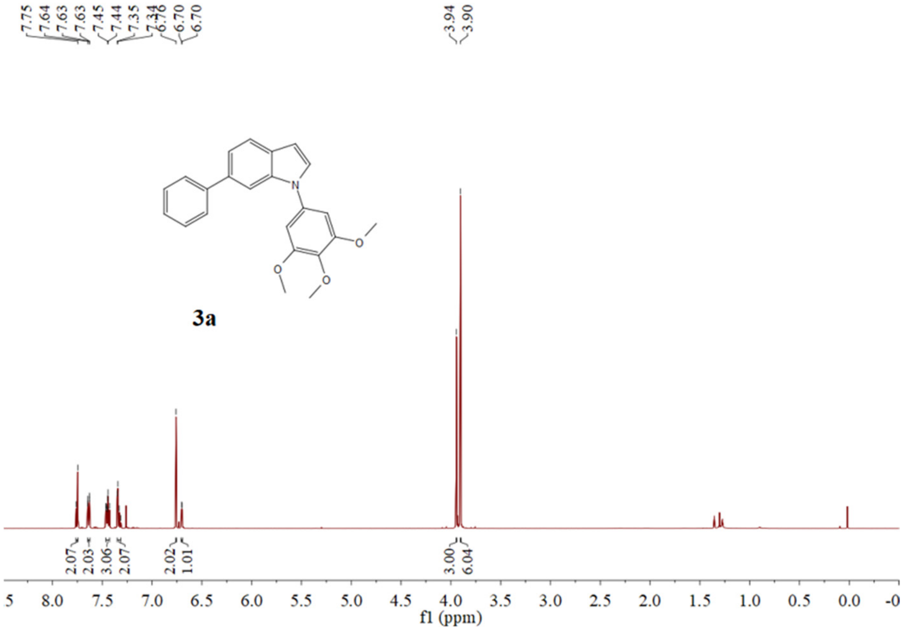

Figure S1A. ^{1}H NMR for compound **3a**

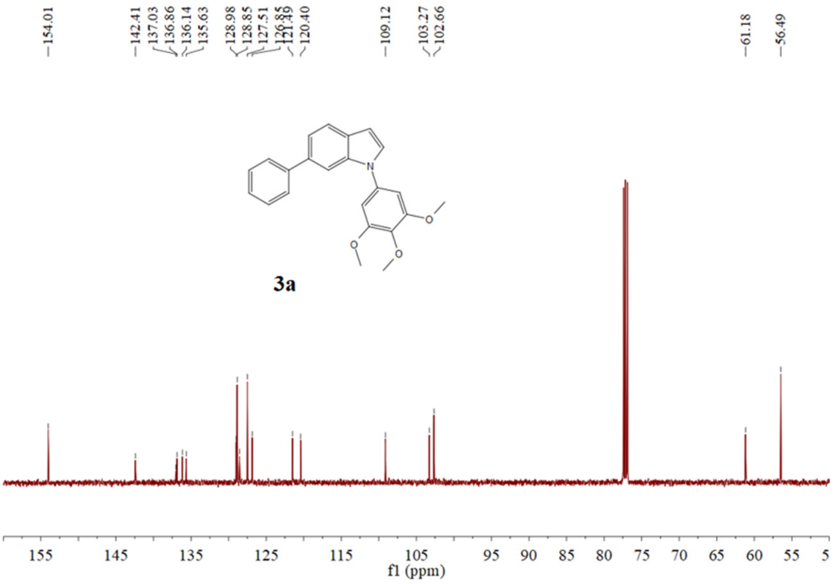

Figure S1B. ^{13}C NMR for compound **3a**

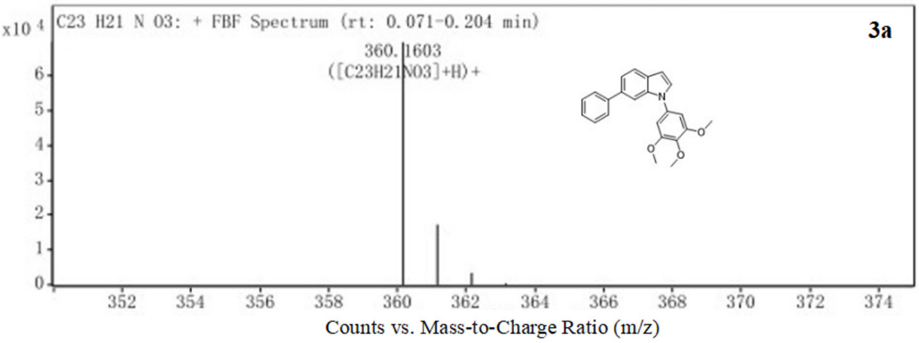

Figure S1C. HRMS for compound **3a**

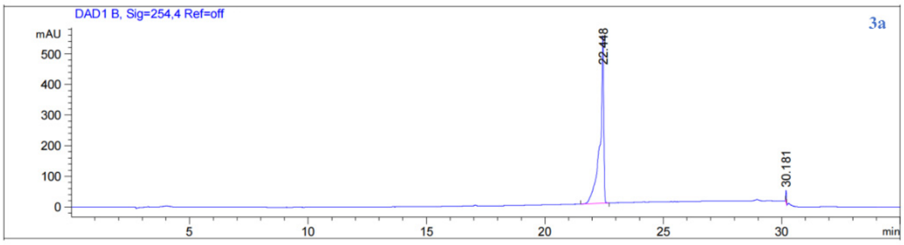

Signal 2: DAD1 B, Sig=254,4 Ref=off

Peak #	Retention time [min]	Type	Peak width [min]	Peak area [mAU*s]	Peak height [mAU]	Peak area %
1	22.448	BB	0.1477	5981.05615	546.52875	98.6112
2	30.181	MM	0.0362	84.23489	38.77095	1.3888

Total amount : 6065.29105 585.29969

Figure S1D. HPLC for compound **3a**

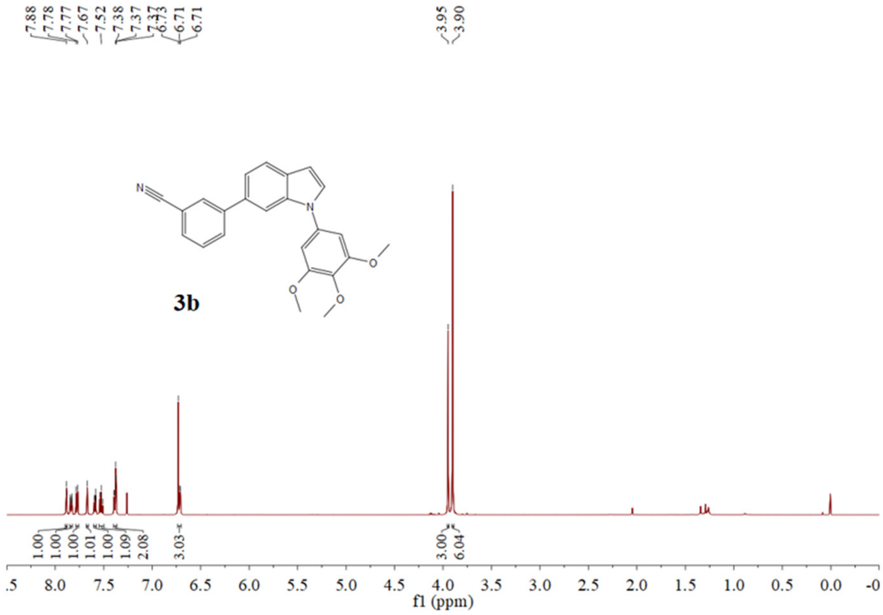

Figure S2A. ^{1}H NMR for compound **3b**

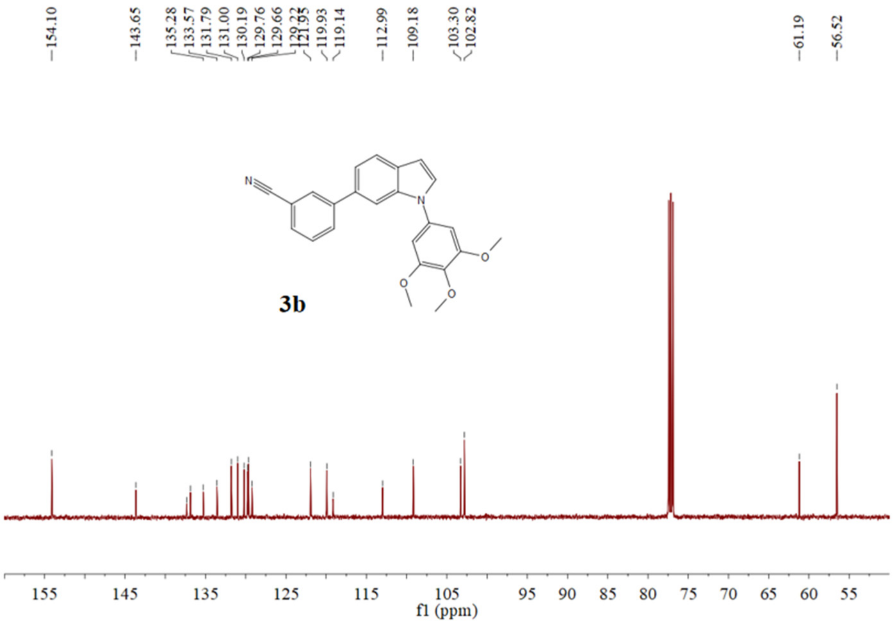

Figure S2B. ^{13}C NMR for compound **3b**

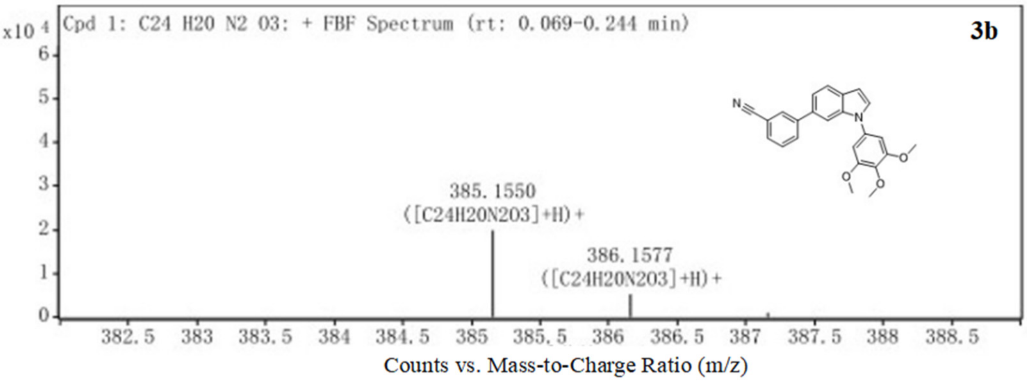

Figure S2C. HRMS for compound **3b**

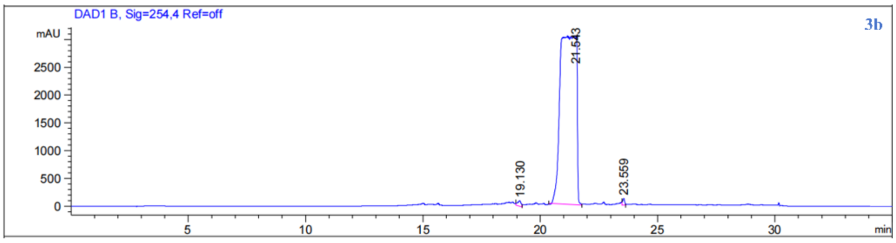

Signal 2: DAD1 B, Sig=254,4 Ref=off

Peak #	Retention time [min]	Type	Peak width [min]	Peak area [mAU*s]	Peak height [mAU]	Peak area %
1	19.130	MM	0.1672	1053.80725	105.05677	0.7090
2	21.543	MM	0.8039	1.46868e5	3044.94678	98.8102
3	23.559	MM	0.0986	714.74133	120.76093	0.4809

Total amount ： 1.48637e5 3270.76448

Figure S2D. HPLC for compound **3b**

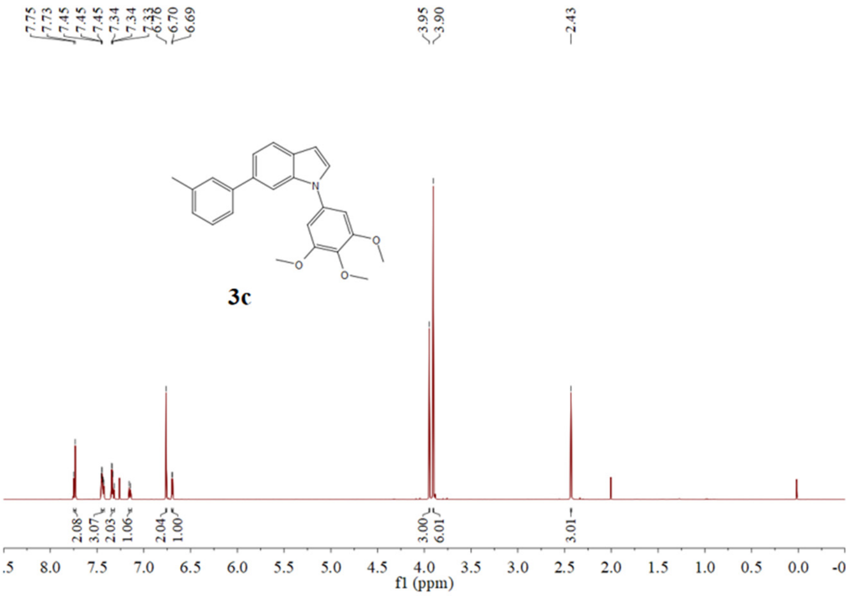

Figure S3A. ^{1}H NMR for compound **3c**

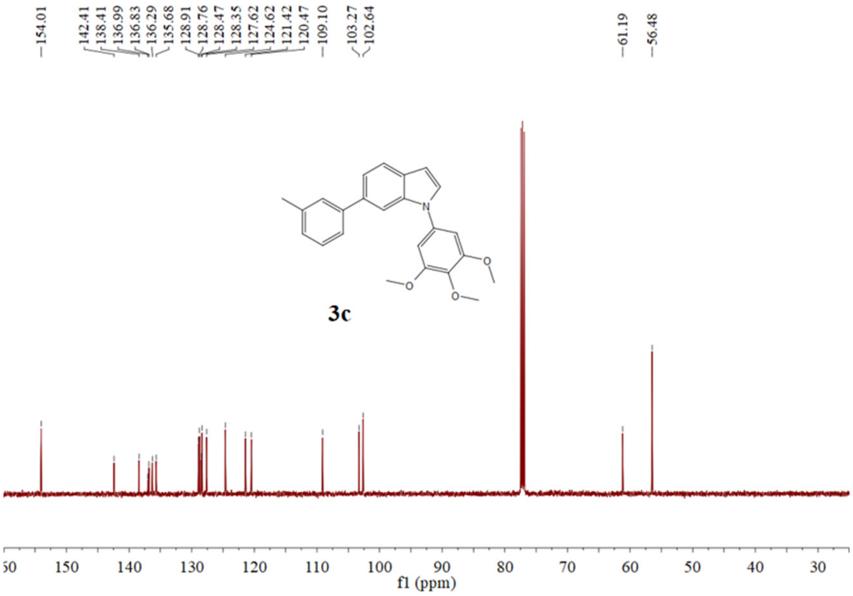

Figure S3B. ^{13}C NMR for compound **3c**

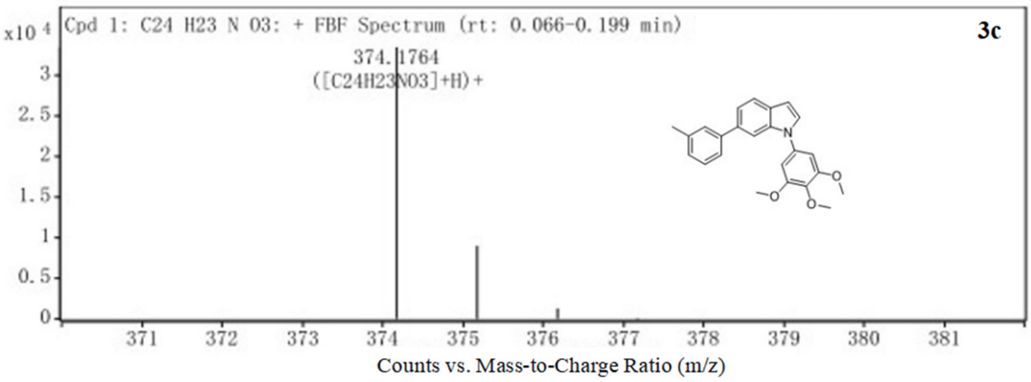

Figure S3C. HRMS for compound **3c**

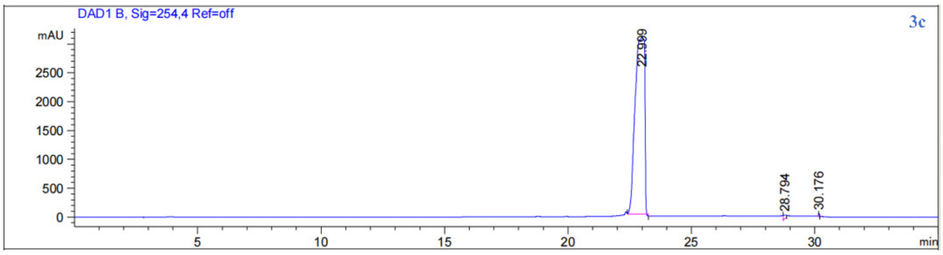

Signal 2: DAD1 B, Sig=254,4 Ref=off

Peak #	Retention time [min]	Type	Peak width [min]	Peak area [mAU*s]	Peak height [mAU]	Peak area %
1	22.999	MM	0.4471	8.21877e4	3063.53931	99.2796
2	28.794	MM	0.1392	493.69293	59.11060	0.5964
3	30.176	MM	0.0334	102.68873	51.27552	0.1240

Total amount : 8.27841e4 3173.92543

Figure S3D. HPLC for compound **3c**

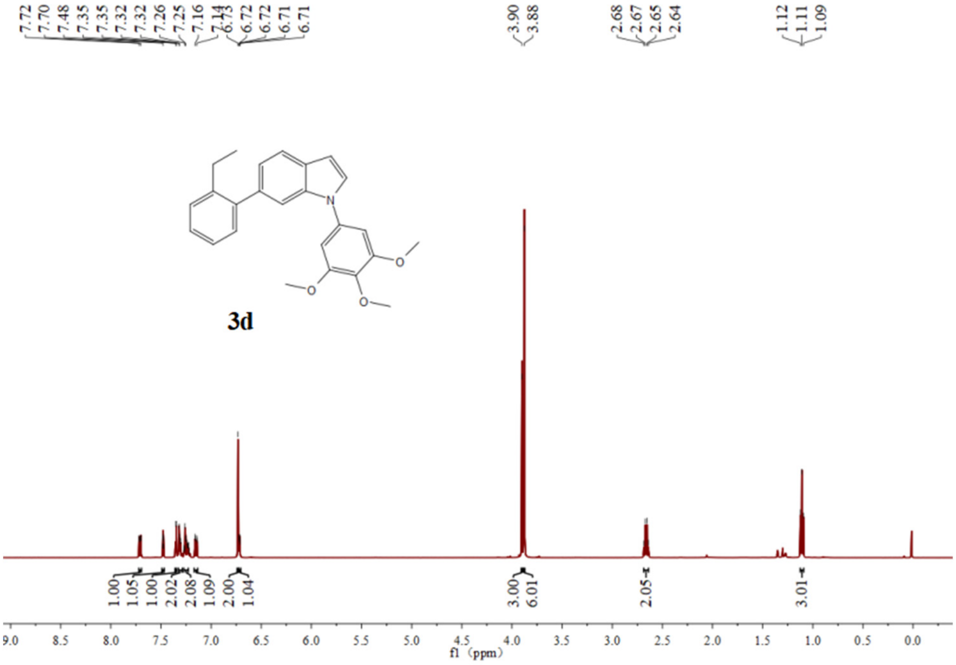

Figure S4A. ^{1}H NMR for compound **3d**

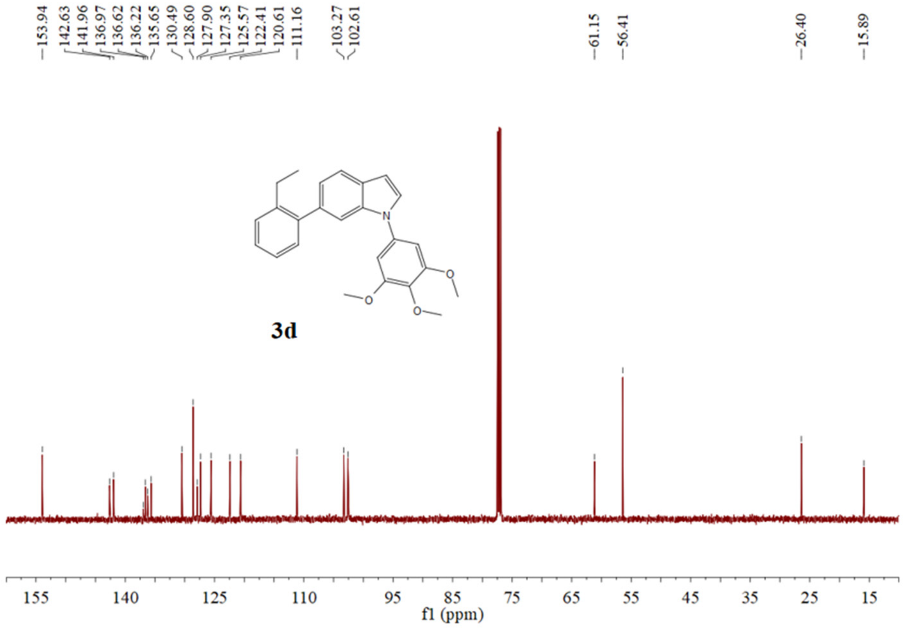

Figure S4B. ^{13}C NMR for compound **3d**

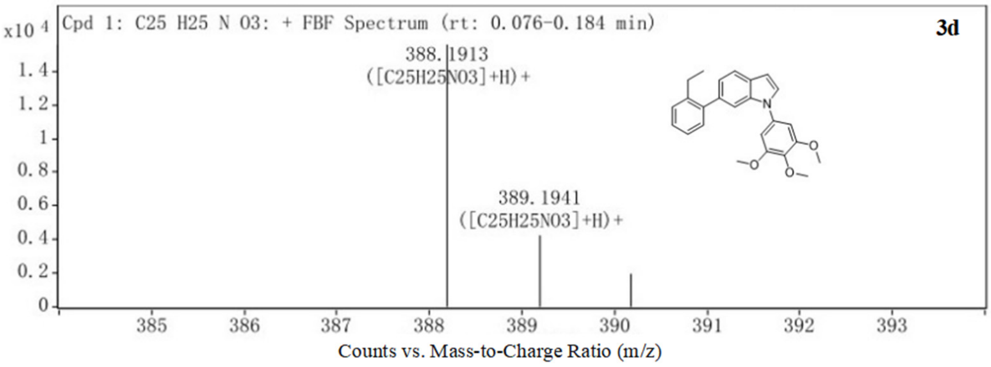

Figure S4C. HRMS for compound **3d**

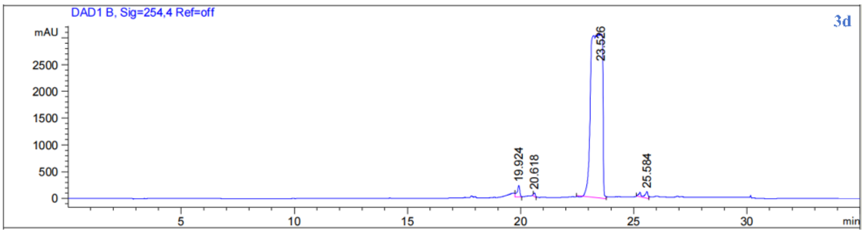

Signal 2: DAD1 B, Sig=254,4 Ref=off

Peak #	Retention time [min]	Type	Peak width [min]	Peak area [mAU*s]	Peak height [mAU]	Peak area %
1	19.924	VB	0.1115	1687.54895	215.26988	1.4885
2	20.618	MM	0.0878	363.89133	69.03714	0.3210
3	23.526	MM	0.5971	1.09614e5	3059.81274	96.6828
4	25.584	MM	0.2239	1709.37634	127.25838	1.5077

Total amount : 1.13375e5 3471.37814

Figure S4D. HPLC for compound **3d**

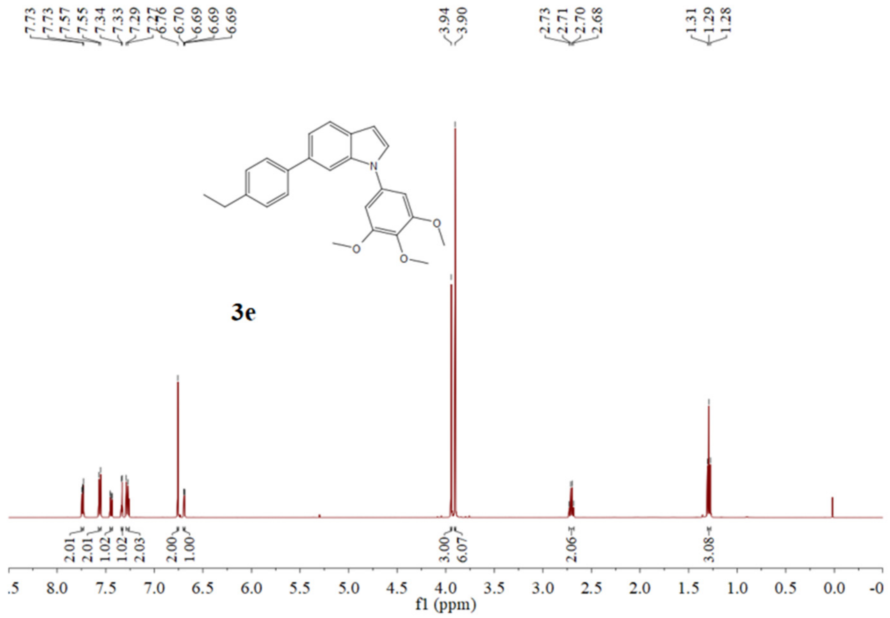

Figure S5A. ^{1}H NMR for compound **3e**

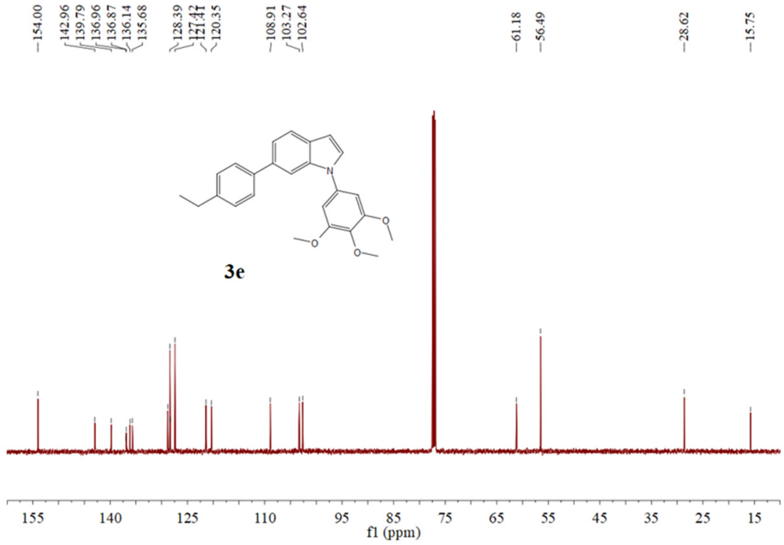

Figure S5B. ^{13}C NMR for compound **3e**

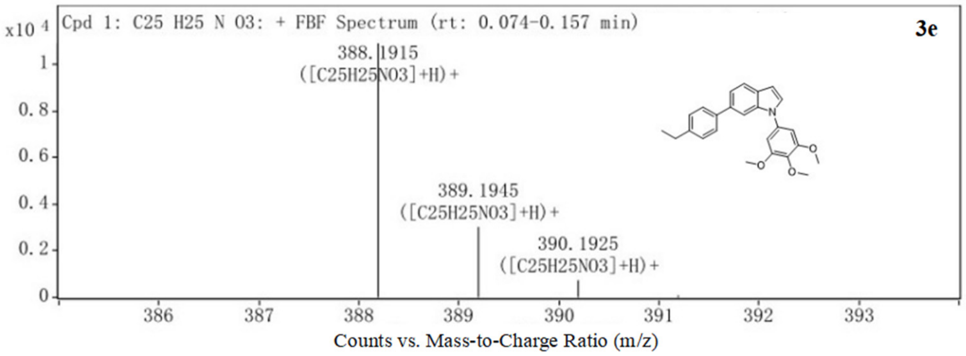

Figure S5C. HRMS for compound **3e**

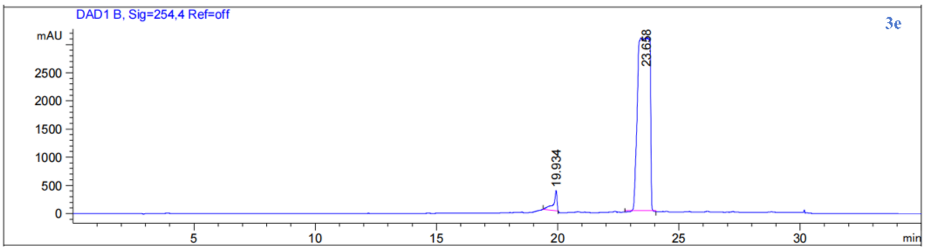

Signal 2: DAD1 B, Sig=254,4 Ref=off

Peak #	Retention time [min]	Type	Peak width [min]	Peak area [mAU*s]	Peak height [mAU]	Peak area %
1	19.934	MM	0.1698	3684.20410	361.66245	3.2959
2	23.658	MM	0.5865	1.08098e5	3071.73975	96.7041

Total amount : 1.11782e5 3433.40219

Figure S5D. HPLC for compound **3e**

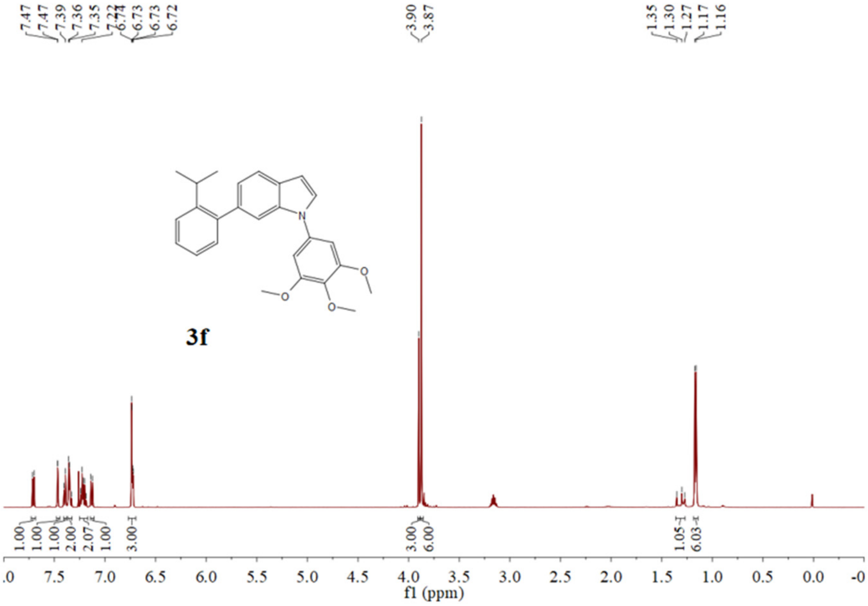

Figure S6A. ^{1}H NMR for compound **3f**

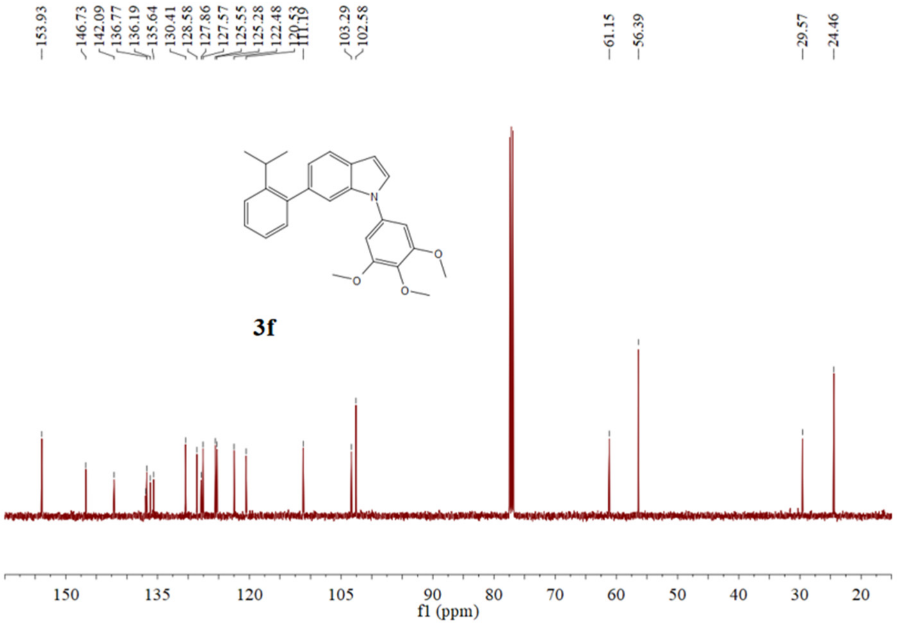

Figure S6B. ^{13}C NMR for compound **3f**

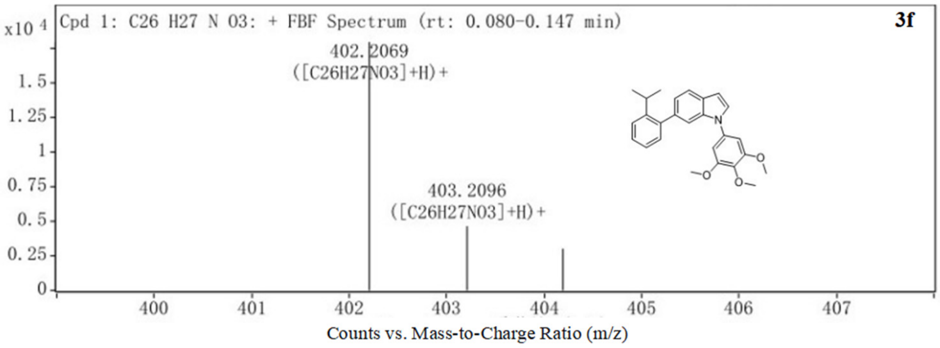

Figure S6C. HRMS for compound **3f**

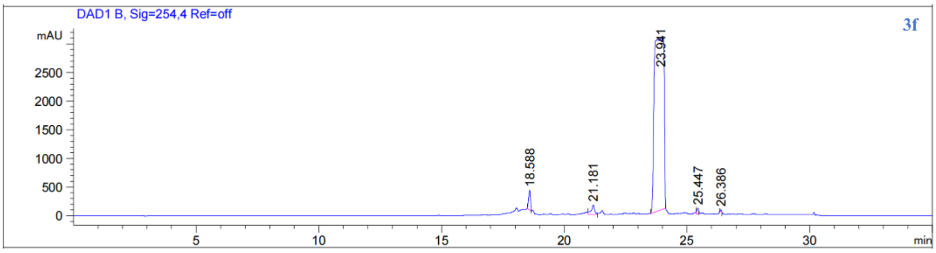

Signal 2: DAD1 B, Sig=254,4 Ref=off

Peak #	Retention time [min]	Type	Peak width [min]	Peak area [mAU*s]	Peak height [mAU]	Peak area %
1	18.588	MM	0.0882	1803.77698	340.90393	2.1152
2	21.181	VV	0.1353	1657.75220	167.76309	1.9439
3	23.941	MM	0.4493	8.12468e4	3014.02490	95.2731
4	25.447	MM	0.0748	460.40762	102.64204	0.5399
5	26.386	MM	0.0522	109.03456	34.78605	0.1279

Total amount : 8.52778e4 3660.12001

Figure S6D. HPLC for compound **3f**

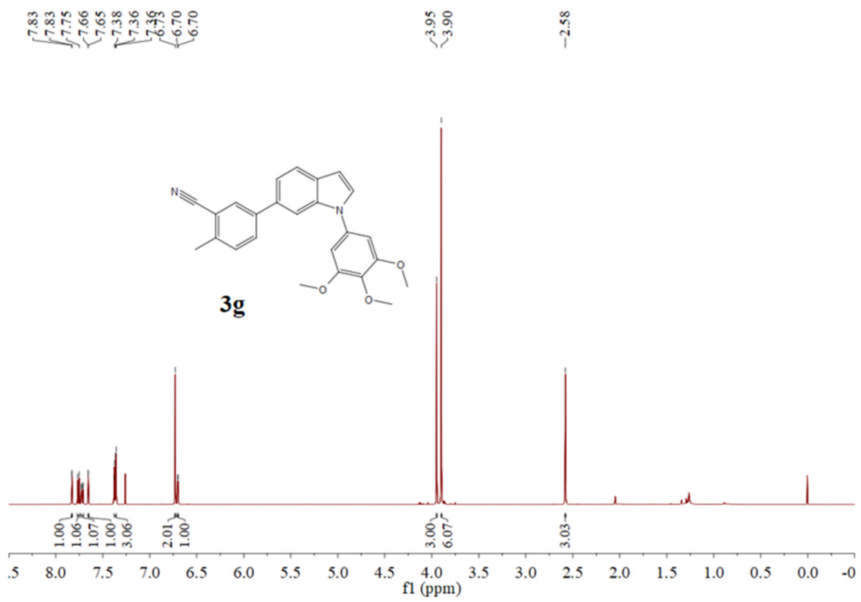

Figure S7A. ^{1}H NMR for compound **3g**

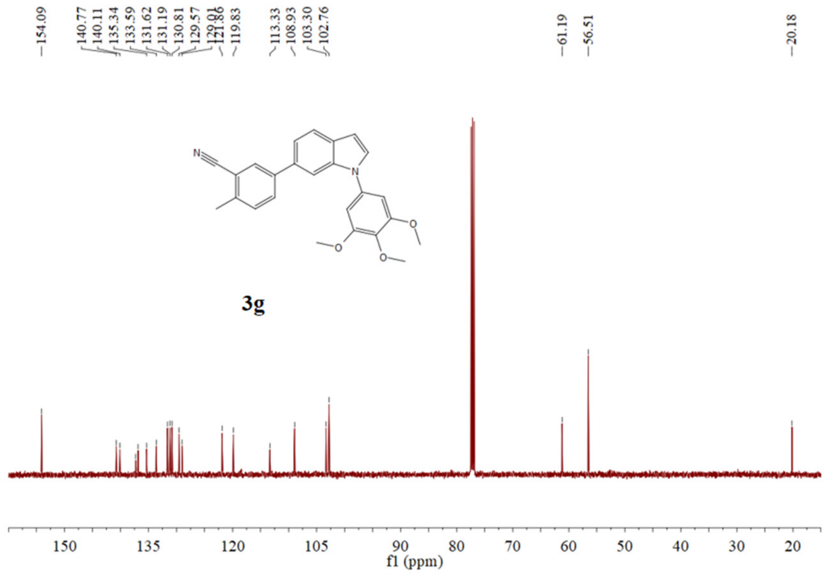

Figure S7B. ^{13}C NMR for compound 3g

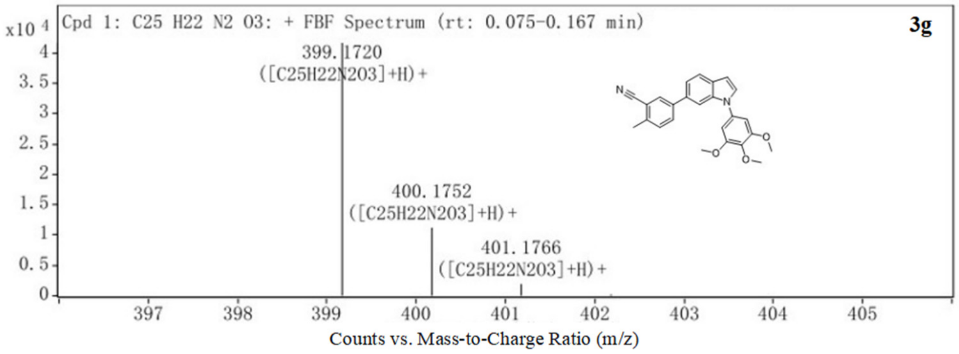

Figure S7C. HRMS for compound **3g**

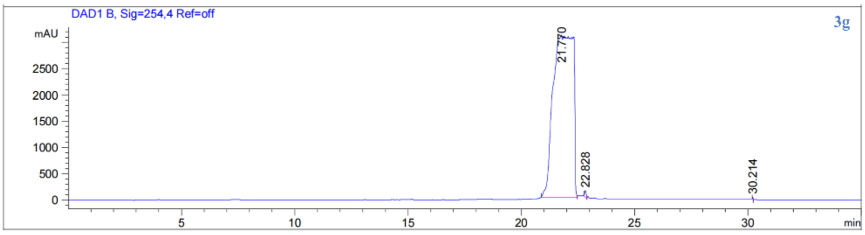

Signal 2: DAD1 B, Sig=254,4 Ref=off

Peak #	Retention time [min]	Type	Peak width [min]	Peak area [mAU*s]	Peak height [mAU]	Peak area %
1	21.770	MM	1.0342	1.91386e5	3084.36353	99.6231
2	22.828	MM	0.0827	639.72760	128.97041	0.3330
3	30.214	MM	0.0386	84.34508	36.38911	0.0439

Total amount : 1.92110e5 3249.72305

Figure S7D. HPLC for compound **3g**

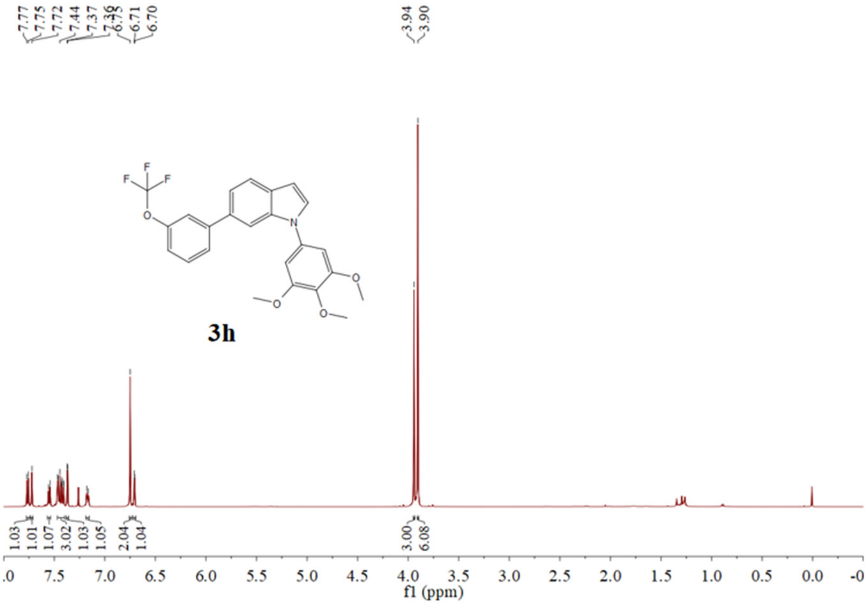

Figure S8A. ^{1}H NMR for compound **3h**

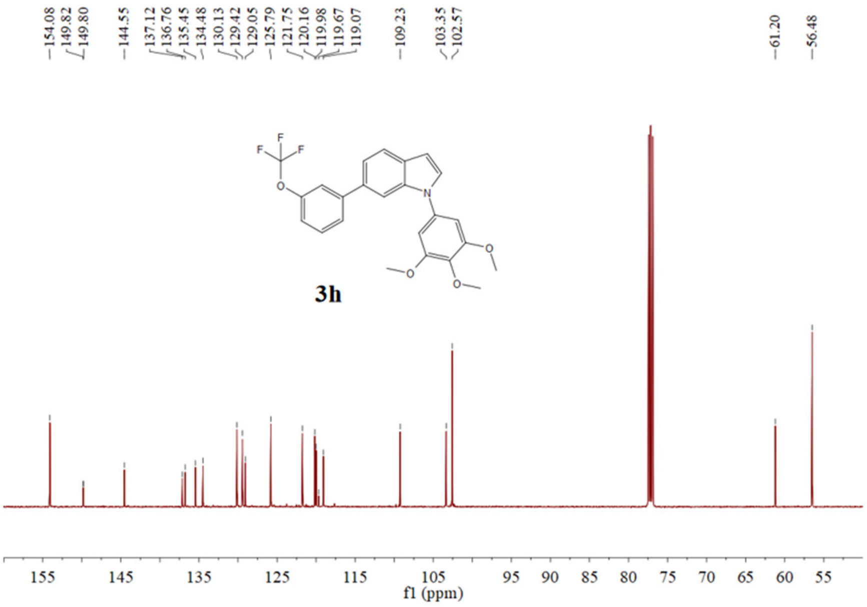

Figure S8B. ^{13}C NMR for compound **3h**

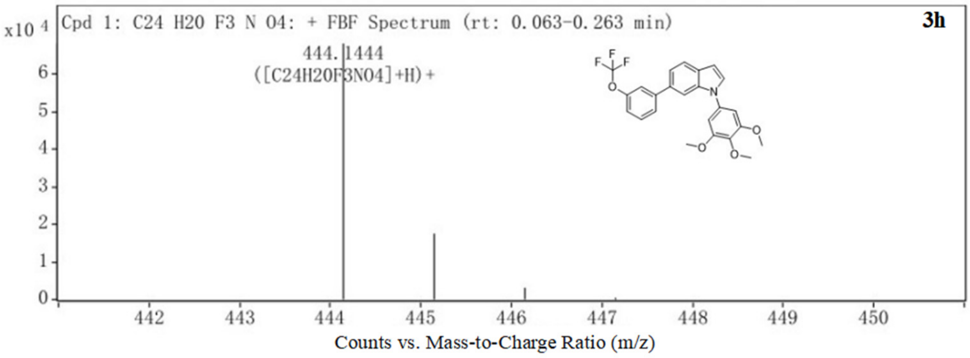

Figure S8C. HRMS for compound **3h**

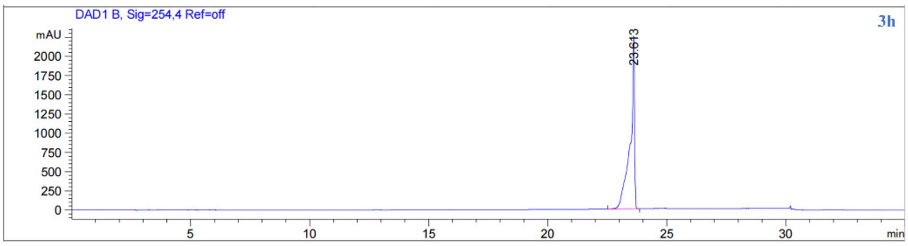

Signal 2: DAD1 B, Sig=254,4 Ref=off

Peak #	Retention time [min]	Type	Peak width [min]	Peak area [mAU*s]	Peak height [mAU]	Peak area %
1	23.613	BB	0.1666	2.81864e4	2242.61646	100.0000

Total amount : 2.81864e4 2242.61646

Figure S8D. HPLC for compound **3h**

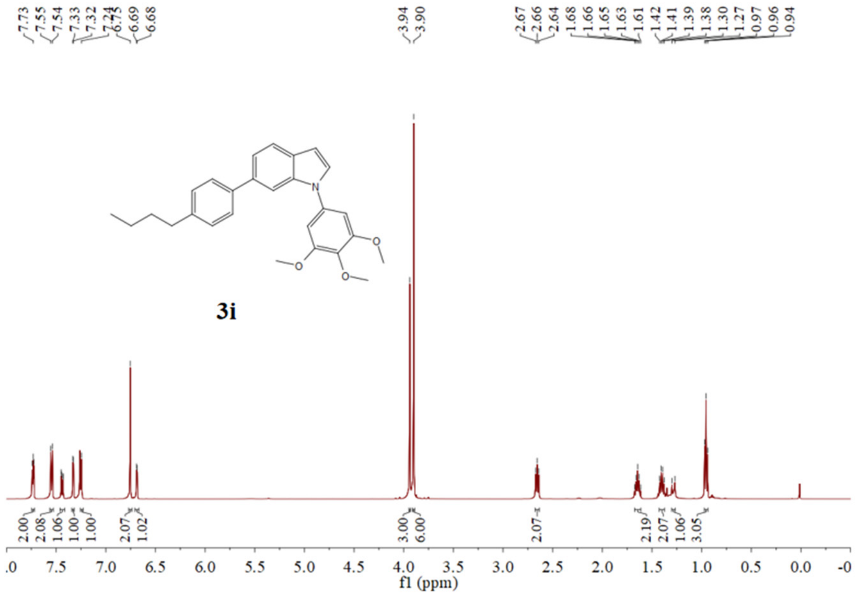

Figure S9A. ^{1}H NMR for compound **3i**

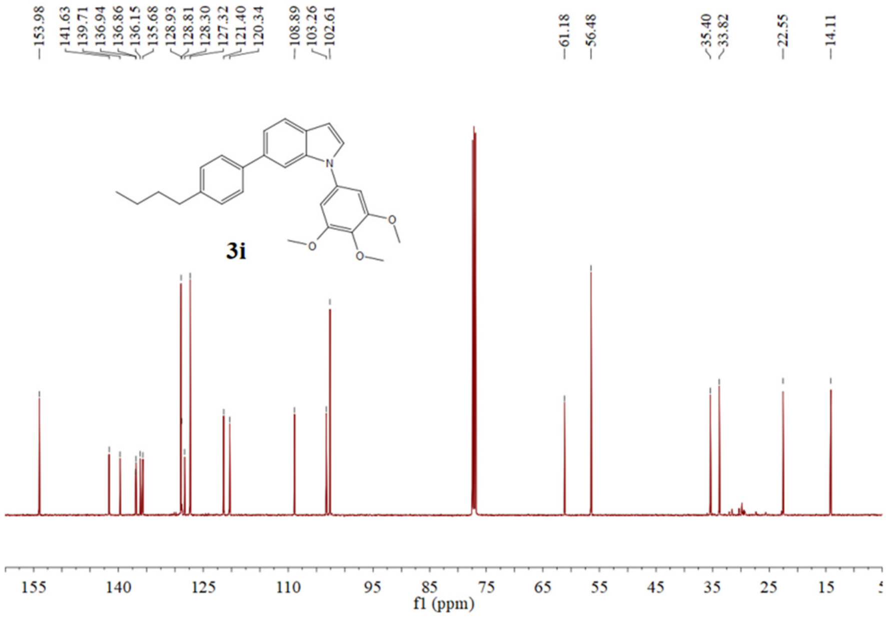

Figure S9B. ^{13}C NMR for compound 3i

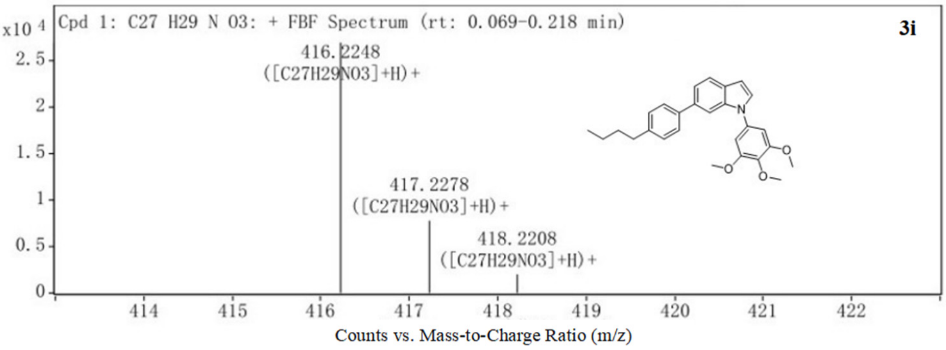

Figure S9C. HRMS for compound **3i**

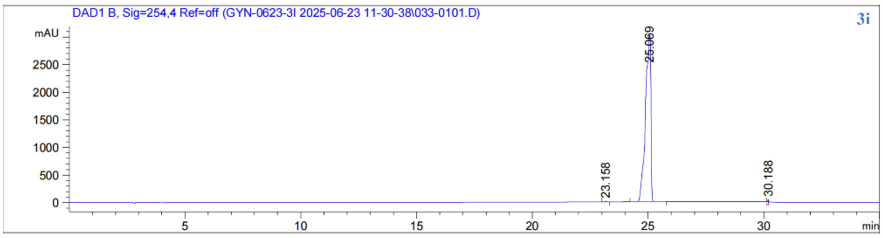

Signal 2: DAD1 B, Sig=254,4 Ref=off

Peak #	Retention time [min]	Type	Peak width [min]	Peak area [mAU*s]	Peak height [mAU]	Peak area %
1	23.158	BB	0.1009	18.79950	2.70954	0.0384
2	25.069	BB	0.2172	4.85712e4	3027.17310	99.1883
3	30.188	MM	0.0624	378.68329	101.07843	0.7733

Total amount : 4.89687e4 3130.96106

Figure S9D. HPLC for compound **3i**

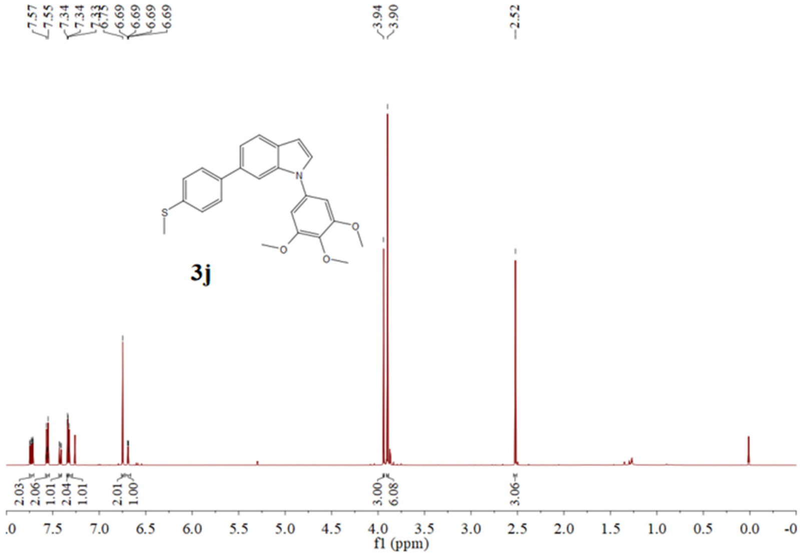

Figure S10A. ^{1}H NMR for compound **3j**

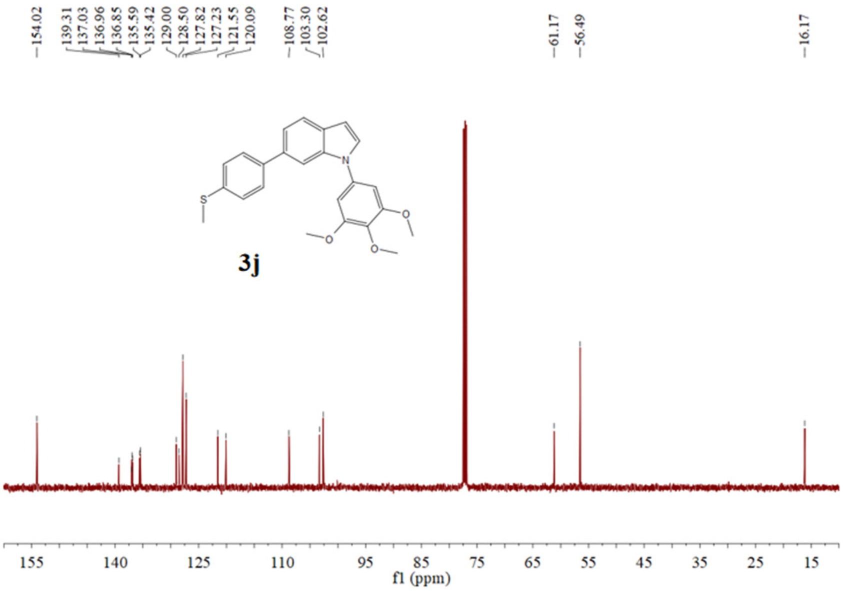

Figure S10B. ^{13}**C NMR for compound 3j**

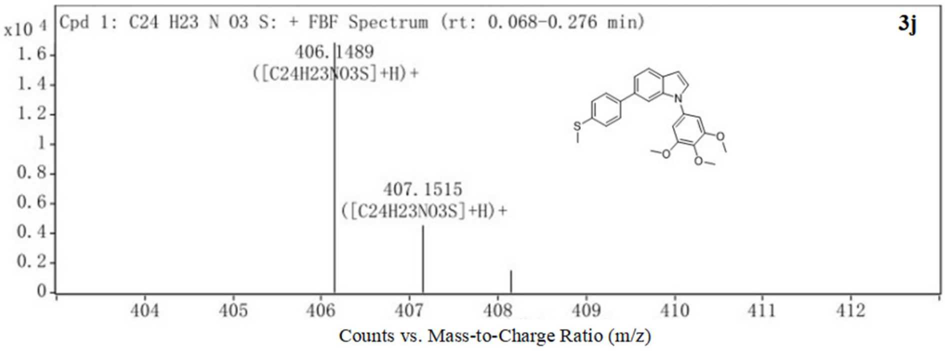

Figure S10C. HRMS for compound **3j**

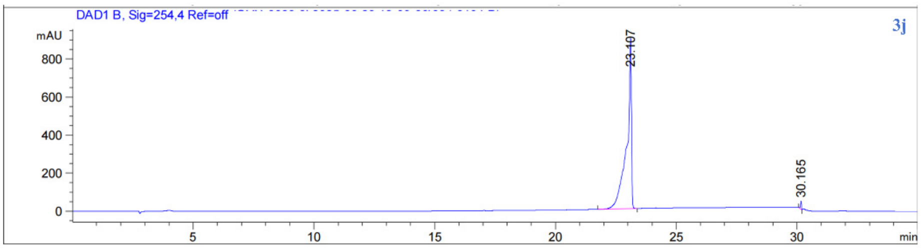

Signal 2: DAD1 B, Sig=254,4 Ref=off

Peak #	Retention time [min]	Type	Peak width [min]	Peak area [mAU*s]	Peak height [mAU]	Peak area %
1	23.107	BB	0.1729	1.17772e4	898.36908	98.9720
2	30.165	BB	0.0448	122.33143	43.51051	1.0280

Total amount : 1.18995e4 941.87959

Figure S10D. HPLC for compound **3j**

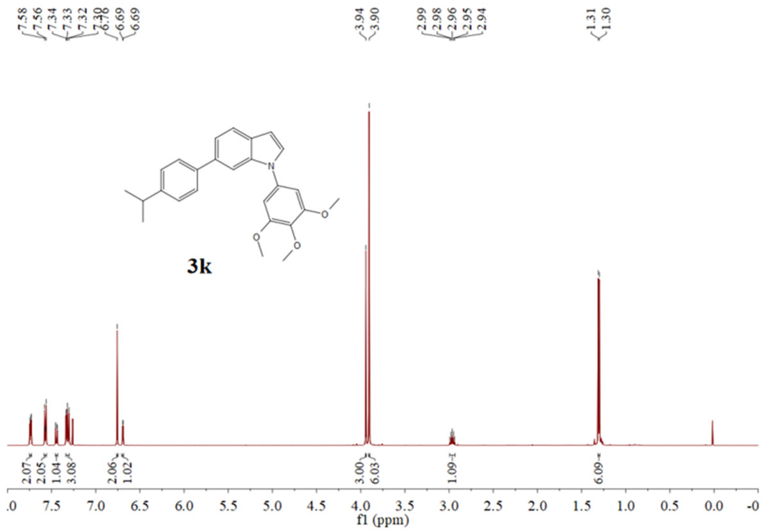

Figure S11A. ^{1}H NMR for compound **3k**

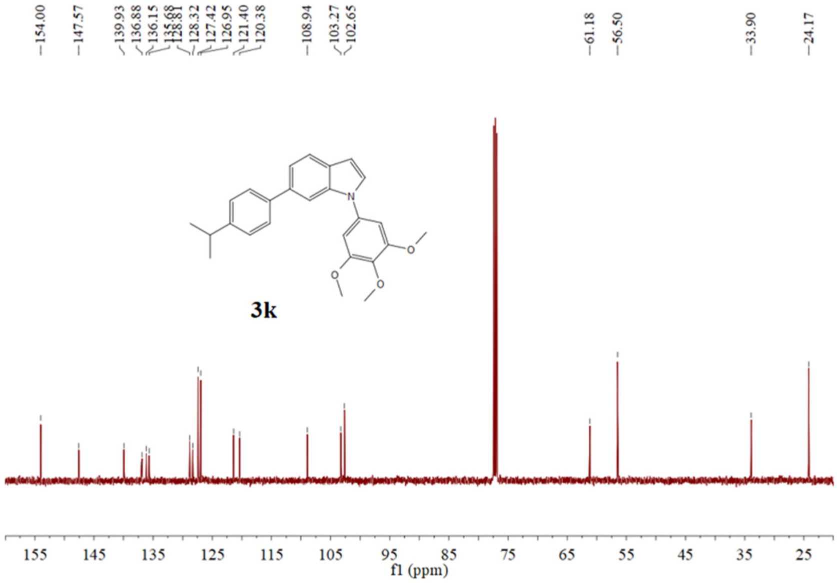

Figure S11B. ^{13}C NMR for compound **3k**

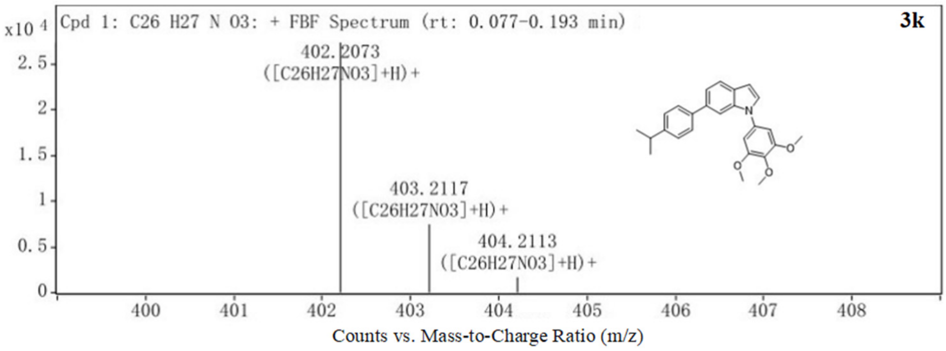

Figure S11C. HRMS for compound **3k**

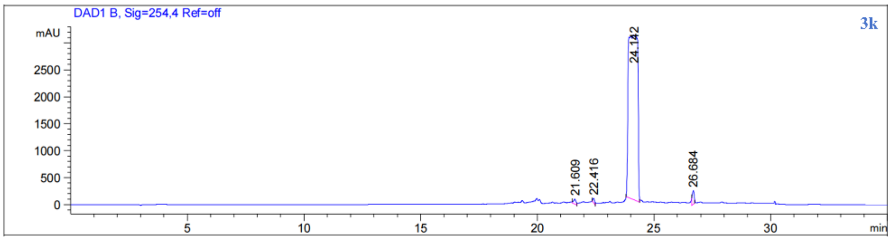

Signal 2: DAD1 B, Sig=254,4 Ref=off

Peak #	Retention time [min]	Type	Peak width [min]	Peak area [mAU*s]	Peak height [mAU]	Peak area %
1	21.609	MM	0.1234	698.25409	94.29043	0.8085
2	22.416	MM	0.0927	468.41733	84.26170	0.5424
3	24.142	MM	0.4561	8.37835e4	3061.35278	97.0123
4	26.684	MM	0.0932	1413.59729	252.83530	1.6368

Total amount : 8.63637e4 3492.74021

Figure S11D. HPLC for compound **3k**

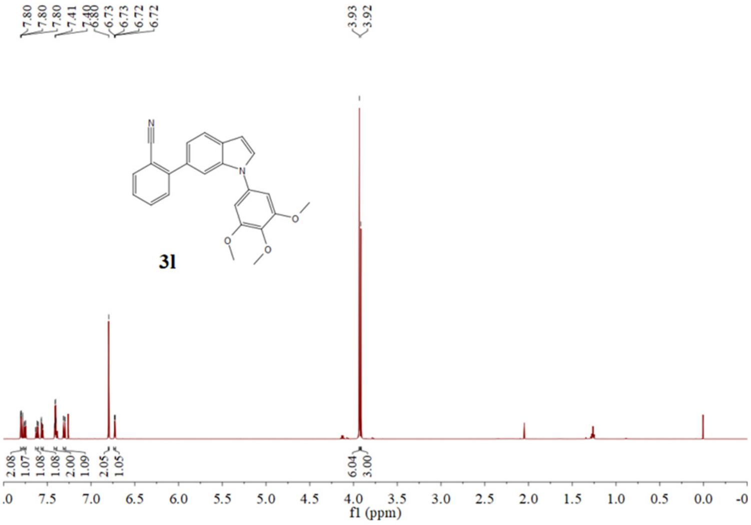

Figure S12A. ^{1}H NMR for compound **3l**

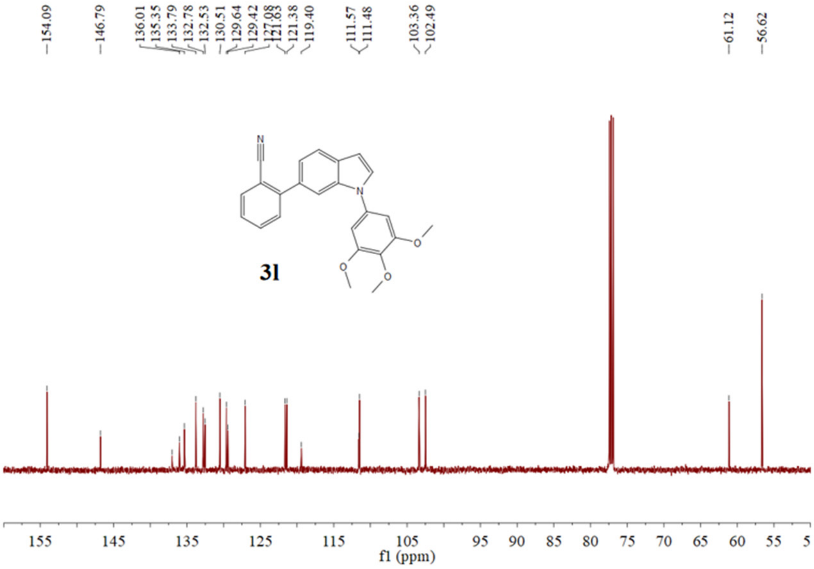

Figure S12B. ^{13}C NMR for compound **3l**

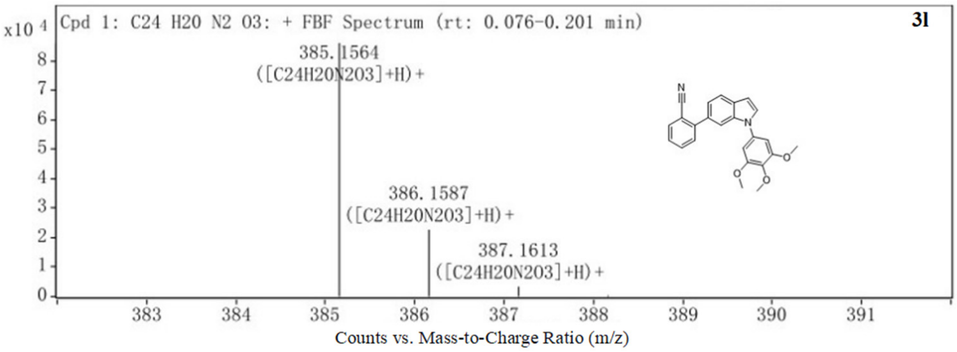

Figure S12C. HRMS for compound **3l**

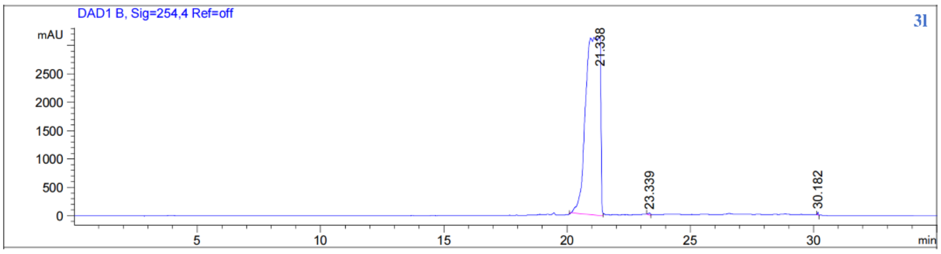

Signal 2: DAD1 B, Sig=254,4 Ref=off

Peak #	Retention time [min]	Type	Peak width [min]	Peak area [mAU*s]	Peak height [mAU]	Peak area %
1	21.338	MM	0.6846	1.29546e5	3153.78931	99.7551
2	23.339	MM	0.1025	208.36923	33.88590	0.1605
3	30.182	MM	0.0400	109.62801	45.66283	0.0844

Total amount : 1.29864e5 3233.33804

Figure S12D. HPLC for compound **3l**

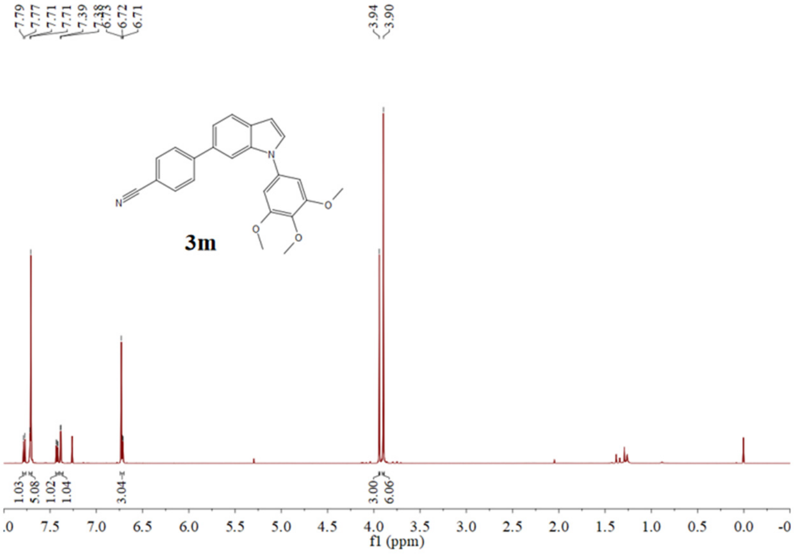

Figure S13A. ^{1}H NMR for compound **3m**

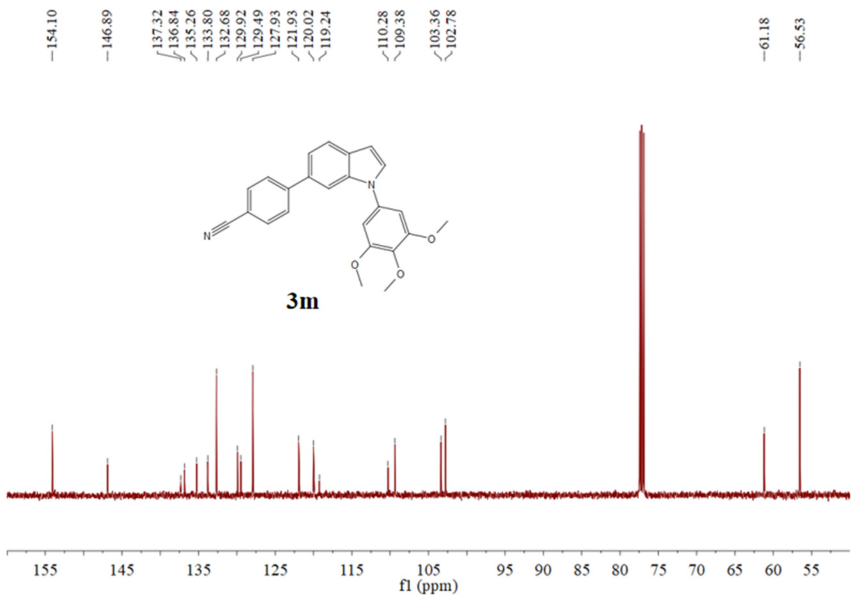

Figure S13B. ^{13}C NMR for compound **3m**

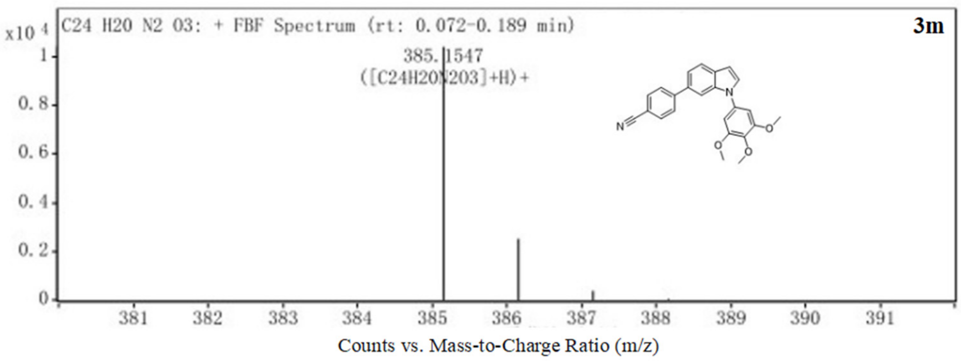

Figure S13C. HRMS for compound **3m**

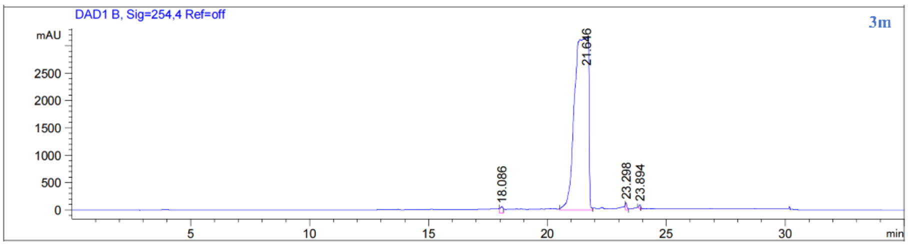

Signal 2: DAD1 B, Sig=254,4 Ref=off

Peak #	Retention time [min]	Type	Peak width [min]	Peak area [mAU*s]	Peak height [mAU]	Peak area %
1	18.086	MM	0.1501	1049.22449	116.47028	0.7976
2	21.646	MM	0.6824	1.29549e5	3163.94995	98.4755
3	23.298	MM	0.0766	589.56873	128.35291	0.4482
4	23.894	MM	0.0750	366.78134	81.45495	0.2788

Total amount : 1.31554e5 3490.22808

Figure S13D. HPLC for compound **3m**

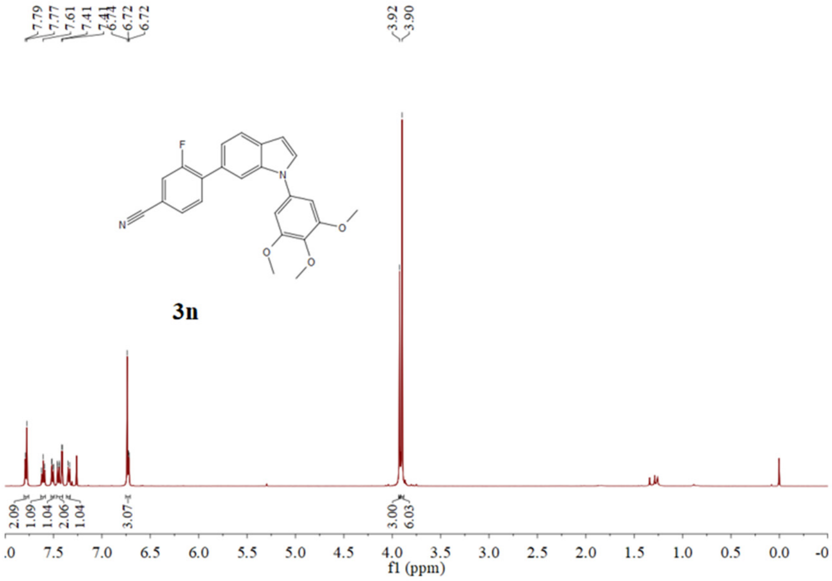

Figure S14A. ^{1}H NMR for compound **3n**

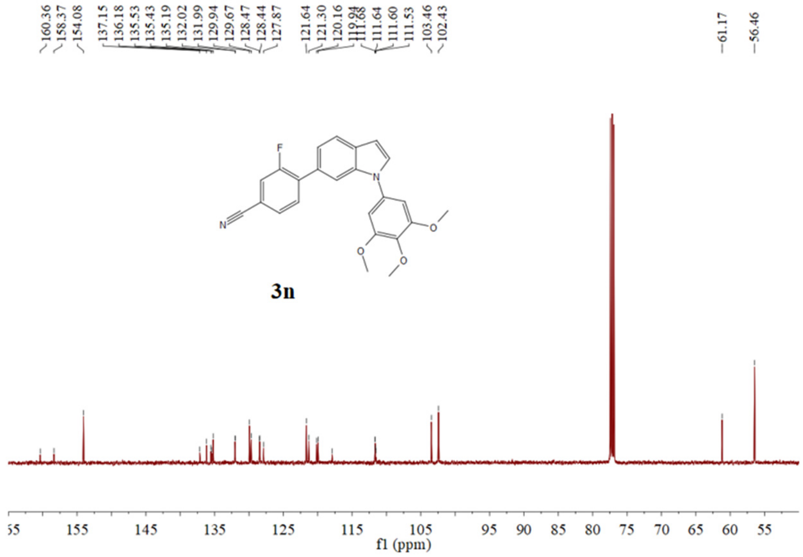

Figure S14B. ^{13}C NMR for compound **3n**

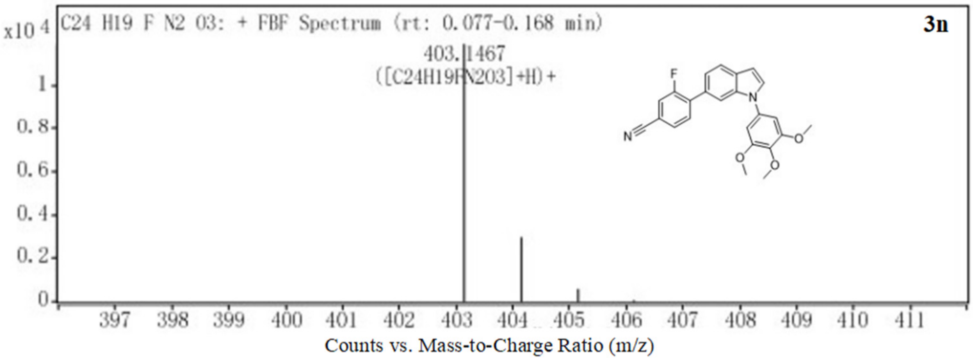

Figure S14C. HRMS for compound **3n**

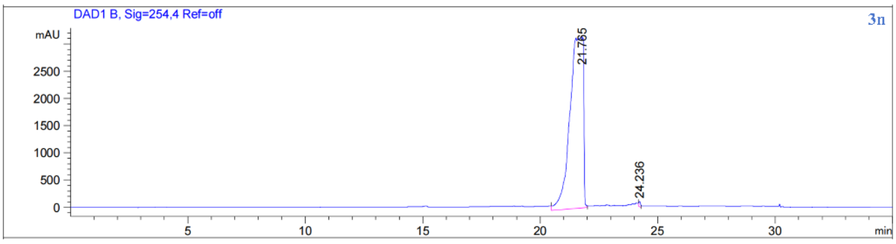

Signal 2: DAD1 B, Sig=254,4 Ref=off

Peak #	Retention time [min]	Type	Peak width [min]	Peak area [mAU*s]	Peak height [mAU]	Peak area %
1	21.765	MM	0.6671	1.26100e5	3150.59985	99.6297
2	24.236	MM	0.0815	468.65533	95.79546	0.3703

Total amount : 1.26568e5 3246.39531

Figure S14D. HPLC for compound **3n**

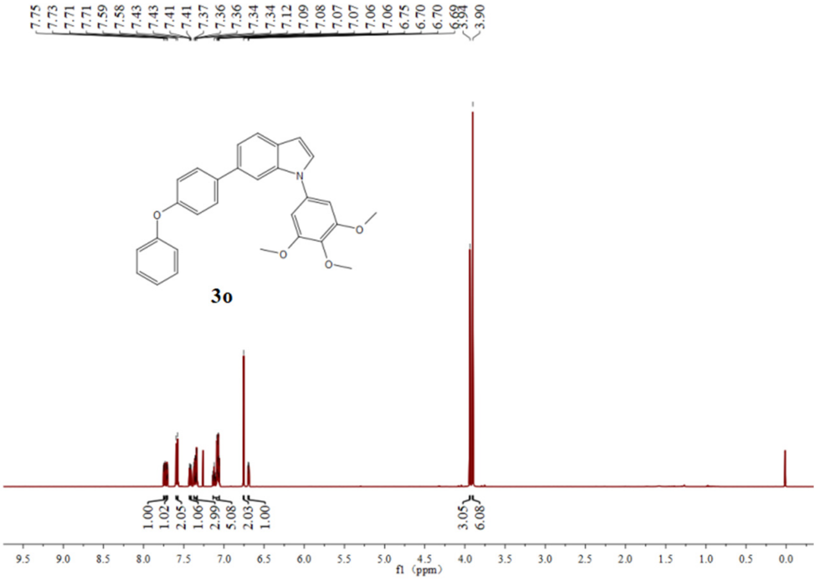

Figure S15A. ^{1}H NMR for compound **3o**

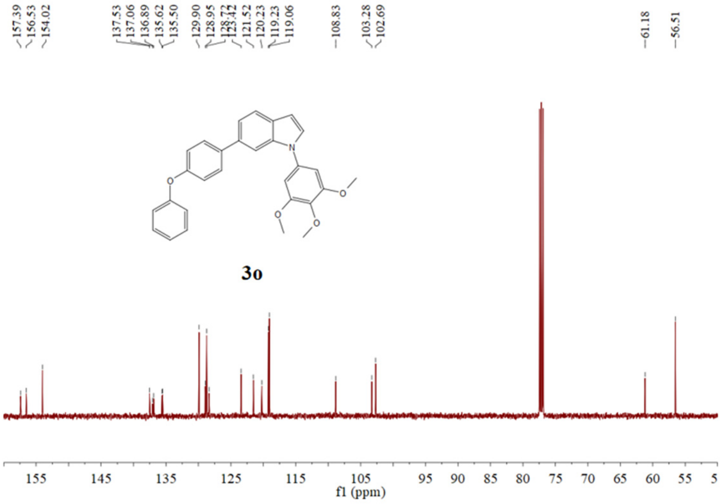

Figure S15B. ^{13}C NMR for compound **3o**

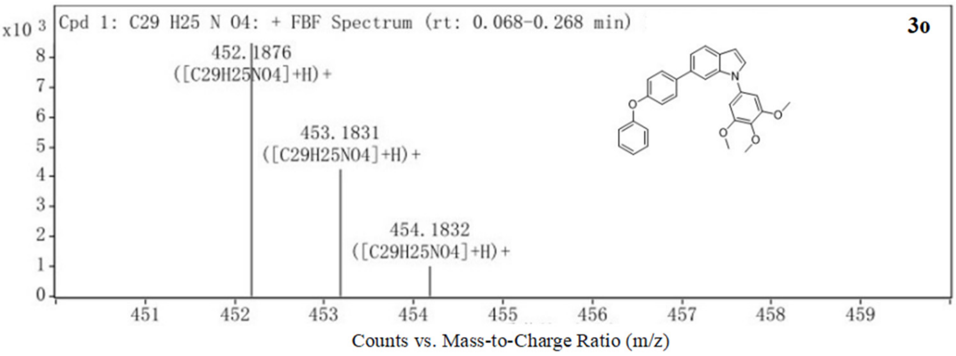

Figure S15C. HRMS for compound **3o**

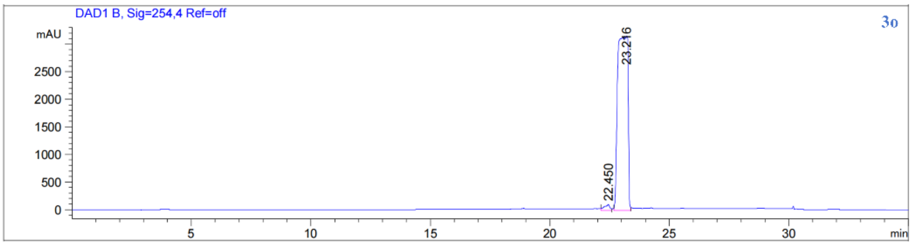

Signal 2: DAD1 B, Sig=254,4 Ref=off

Peak #	Retention time [min]	Type	Peak width [min]	Peak area [mAU*s]	Peak height [mAU]	Peak area %
1	22.450	MM	0.2685	1788.08691	110.99899	1.8617
2	23.216	MM	0.4962	9.42595e4	3166.25366	98.1383

Total amount : 9.60476e4 3277.25265

Figure S15D. HPLC for compound **3o**

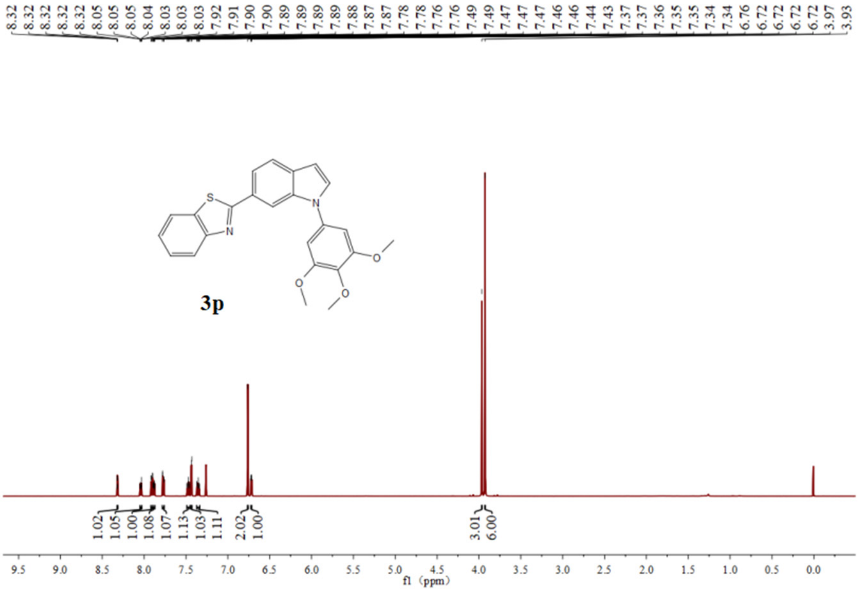

Figure S16A. ^{1}H NMR for compound **3p**

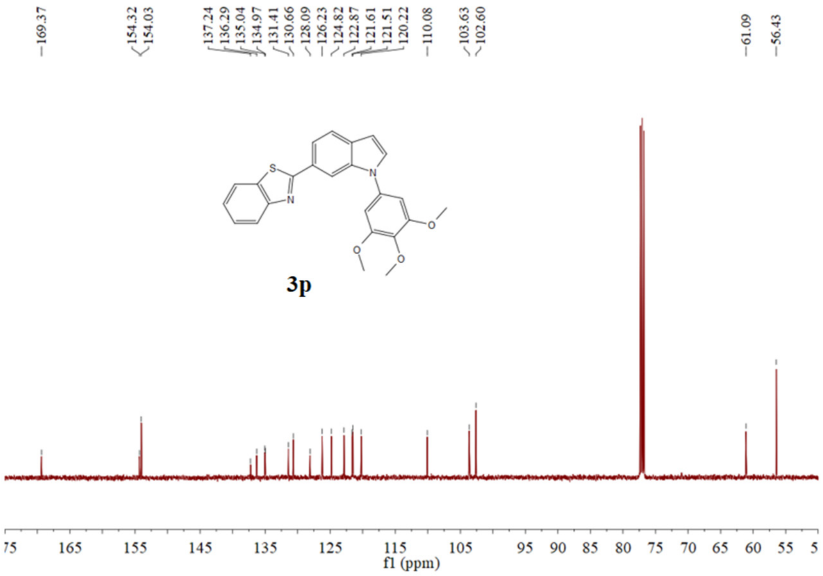

Figure S16B. ^{13}C NMR for compound **3p**

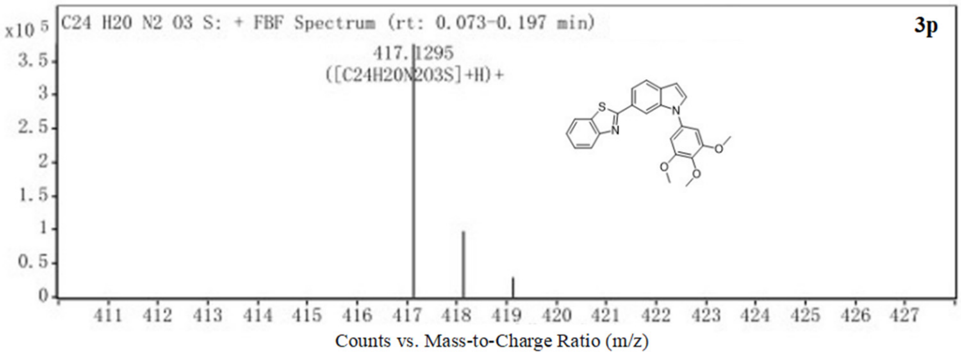

Figure S16C. HRMS for compound **3p**

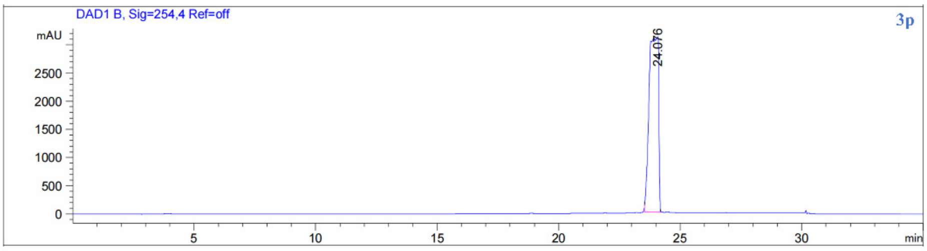

Signal 2: DAD1 B, Sig=254,4 Ref=off

Peak #	Retention time [min]	Type	Peak width [min]	Peak area [mAU*s]	Peak height [mAU]	Peak area %
1	24.076	MM	0.4537	8.44457e4	3101.80420	100.0000

Total amount : 8.44457e4 3101.80420

Figure S16D. HPLC for compound **3p**

Analysis of the affinity of 3a-3g to tubulin by bio-layer interferometry (BLI)

BLI assays were performed on an Octet RED96 instrument (FortéBio, Pall Life Sciences) at 25 °C in PBS buffer supplemented with 0.02% (v/v) Tween-20 as the running buffer. Biotinylated tubulin proteins (3 μg/mL) were immobilized onto Super Streptavidin (SSA) biosensors. Association and dissociation were monitored for 60 s each. Compounds were tested at serial concentrations of 200, 100, 50, 25, 12.5, and 6.25 μM. Reference subtraction was performed using sensors incubated in running buffer without protein loading to correct for baseline drift. Data were analyzed using Octet Data Analysis software (version 9.0), and equilibrium dissociation constants (K_D) were determined by fitting the curves to a 1:1 binding model. All measurements were performed in triplicate, and results are reported as mean ± SD.

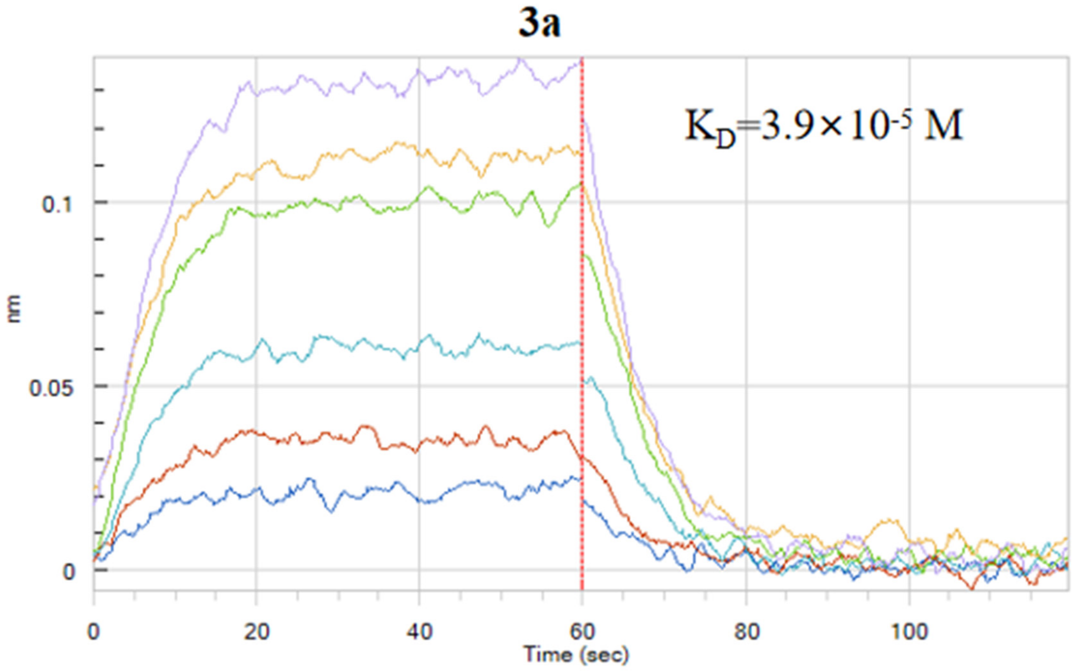

Figure S17. the affinity of compound **3a**

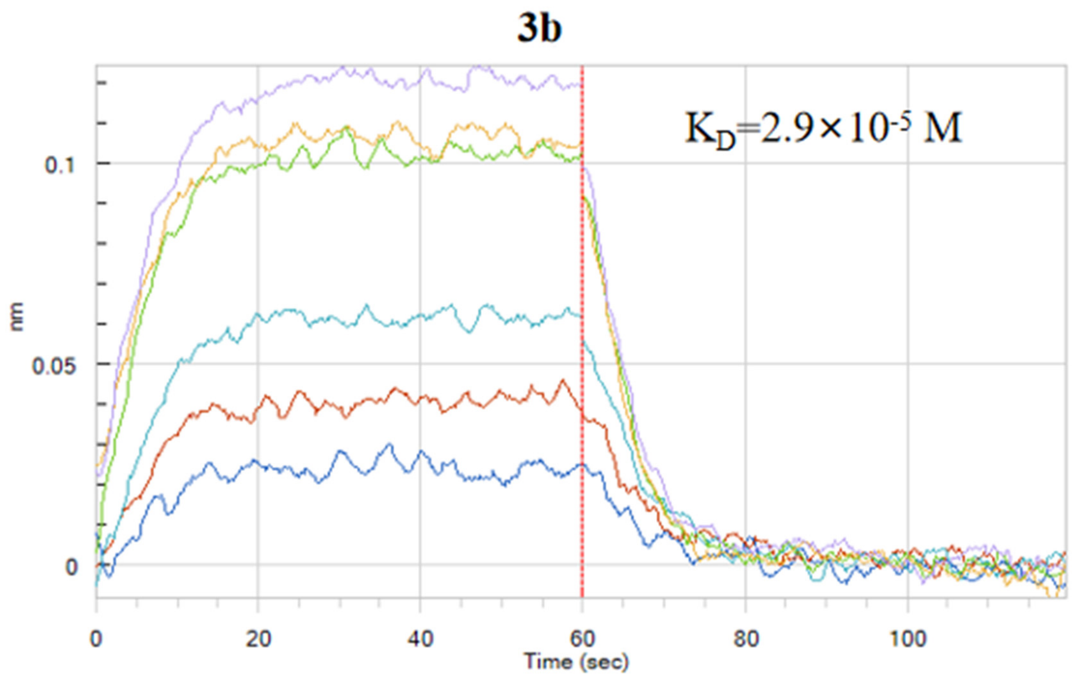

Figure S18. the affinity of compound **3b**

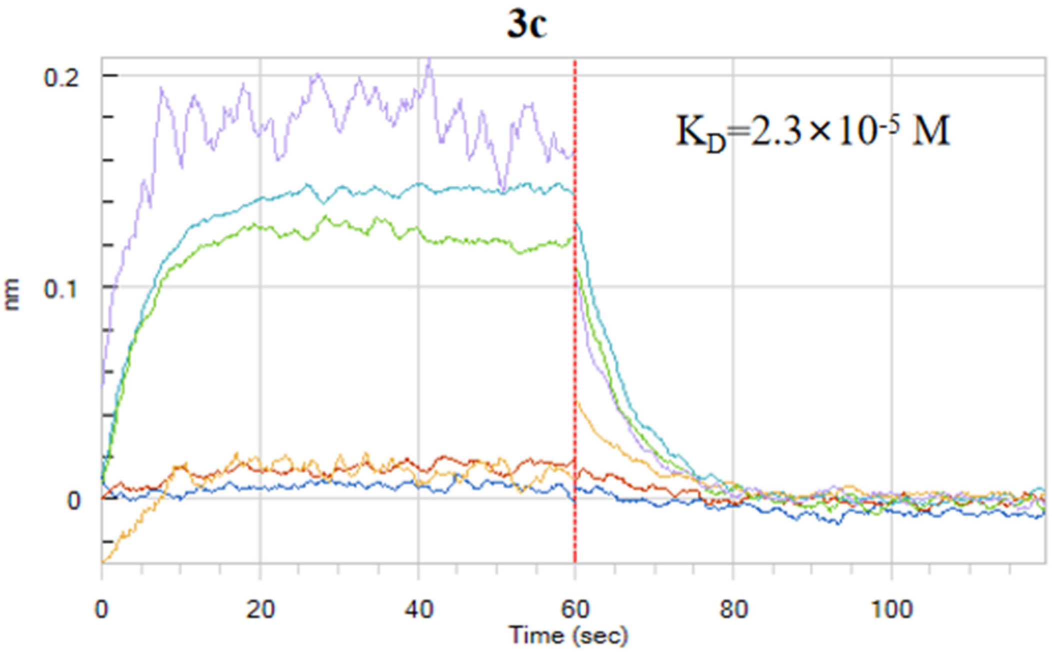

Figure S19. the affinity of compound **3c**

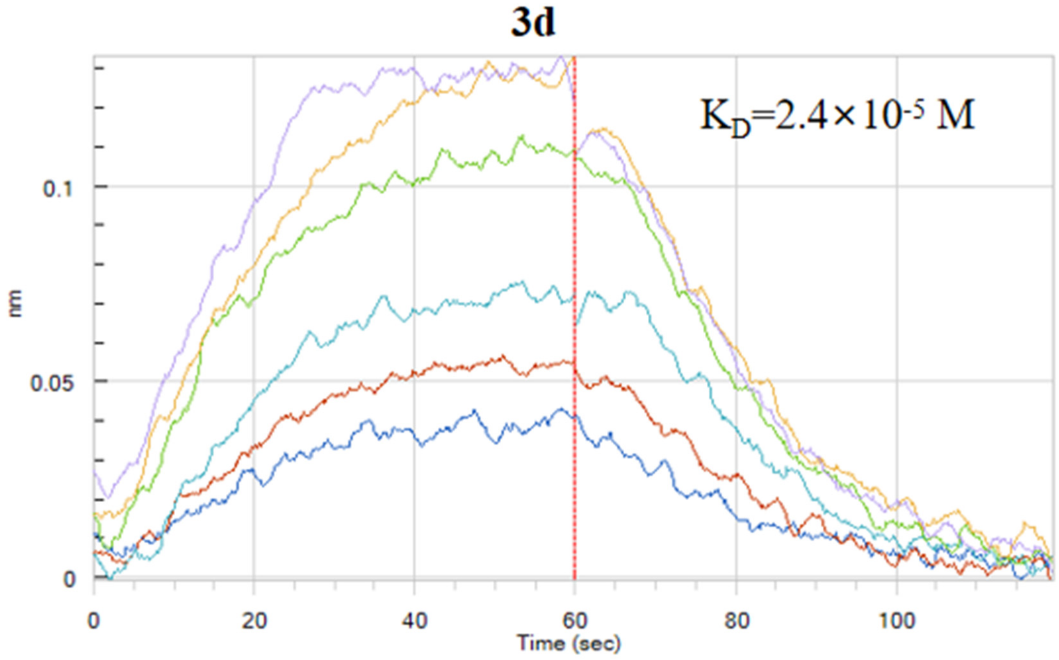

Figure S20. the affinity of compound **3d**

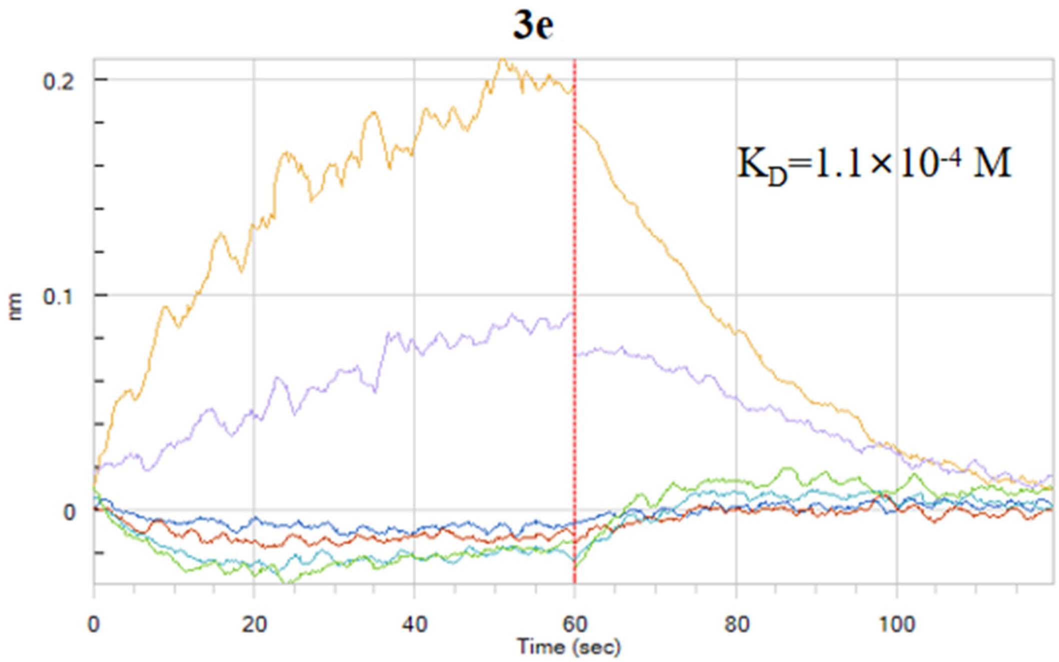

Figure S21. the affinity of compound **3e**

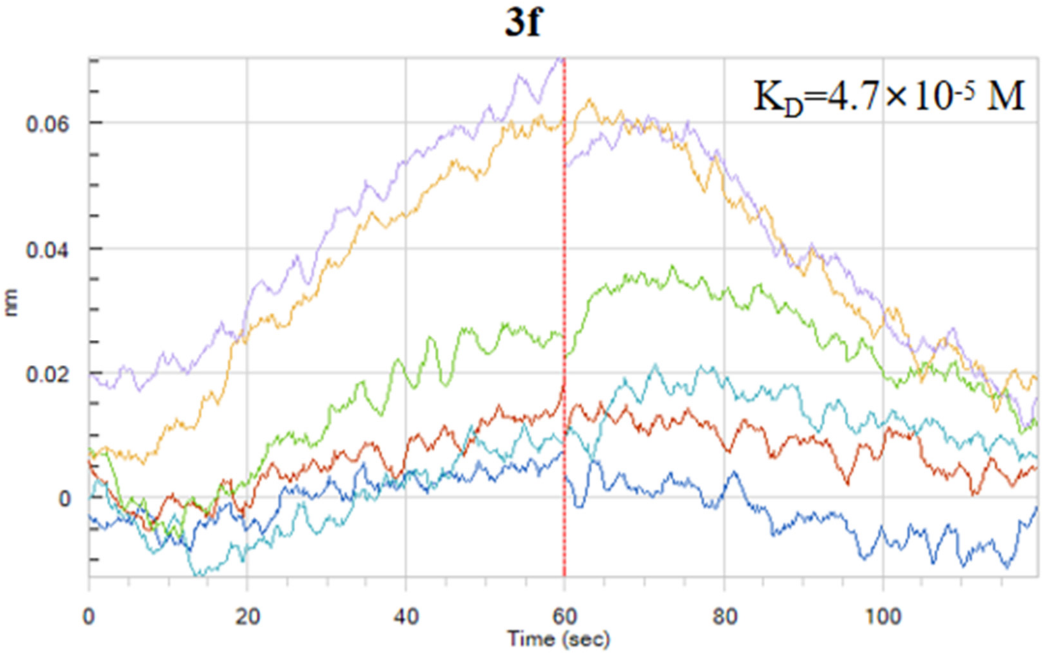

Figure S22. the affinity of compound **3f**

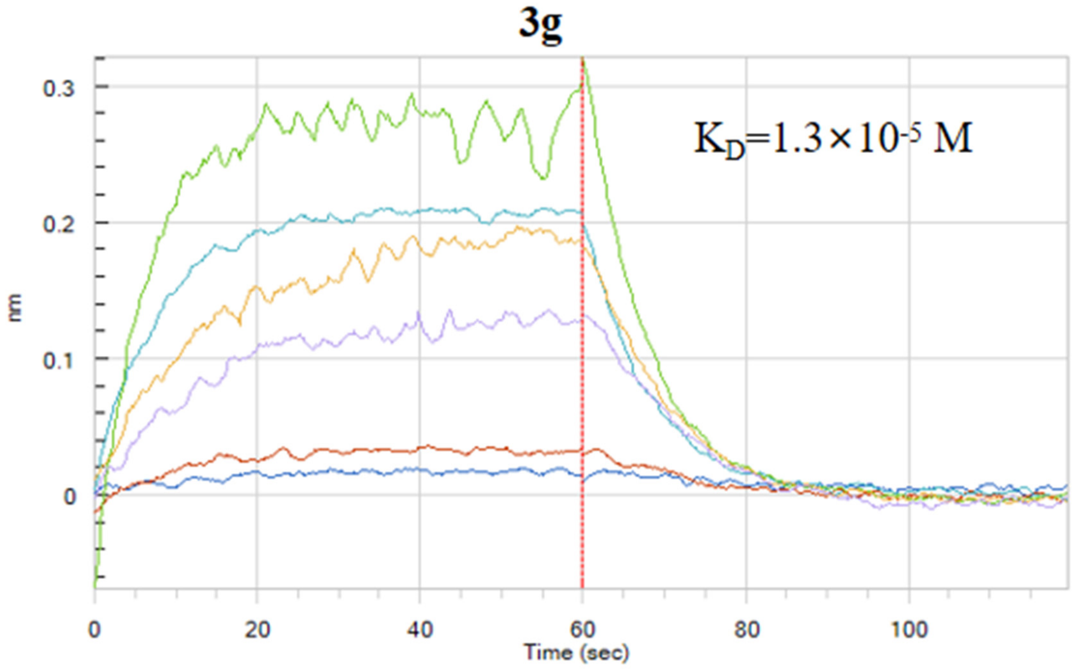

Figure S23. the affinity of compound **3g**

3h

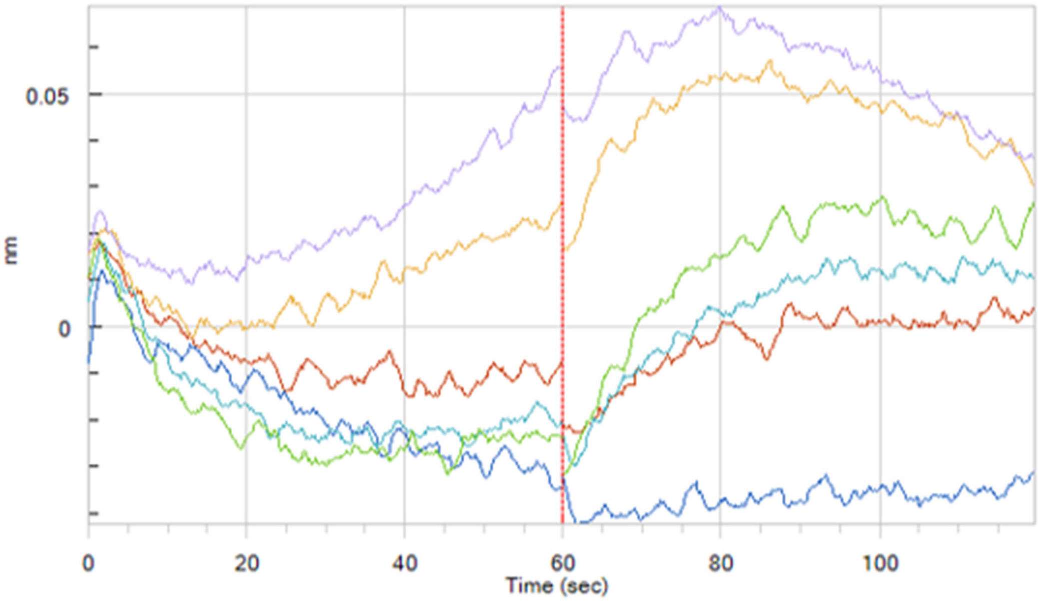

Figure S24. the affinity of compound **3h**

3i

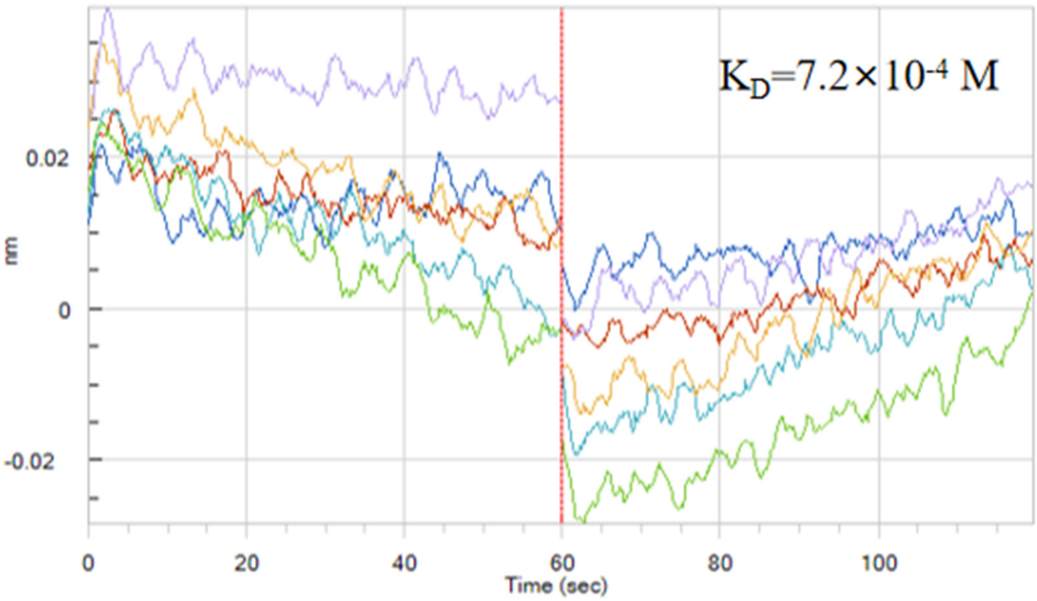

Figure S25. the affinity of compound **3i**

3j

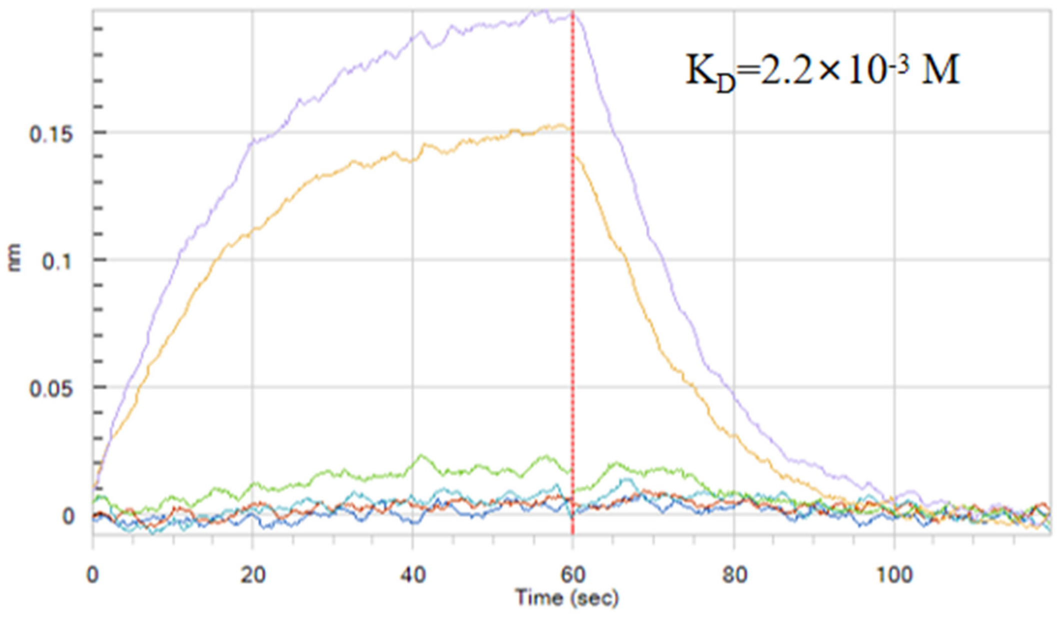

Figure S26. the affinity of compound **3j**

3k

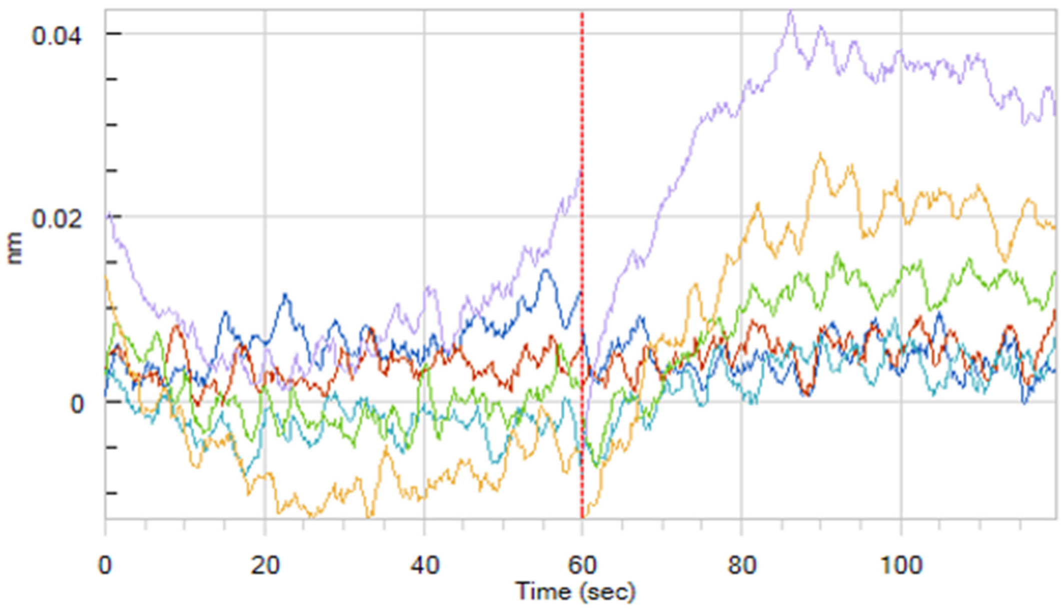

Figure S27. the affinity of compound **3k**

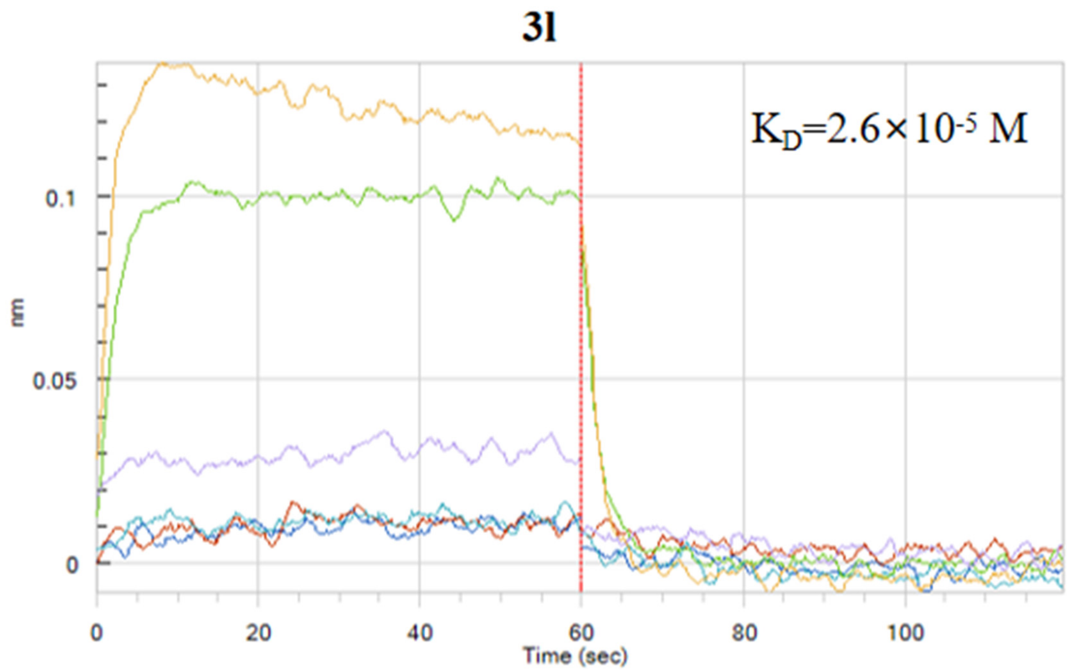

Figure S28. the affinity of compound **3l**

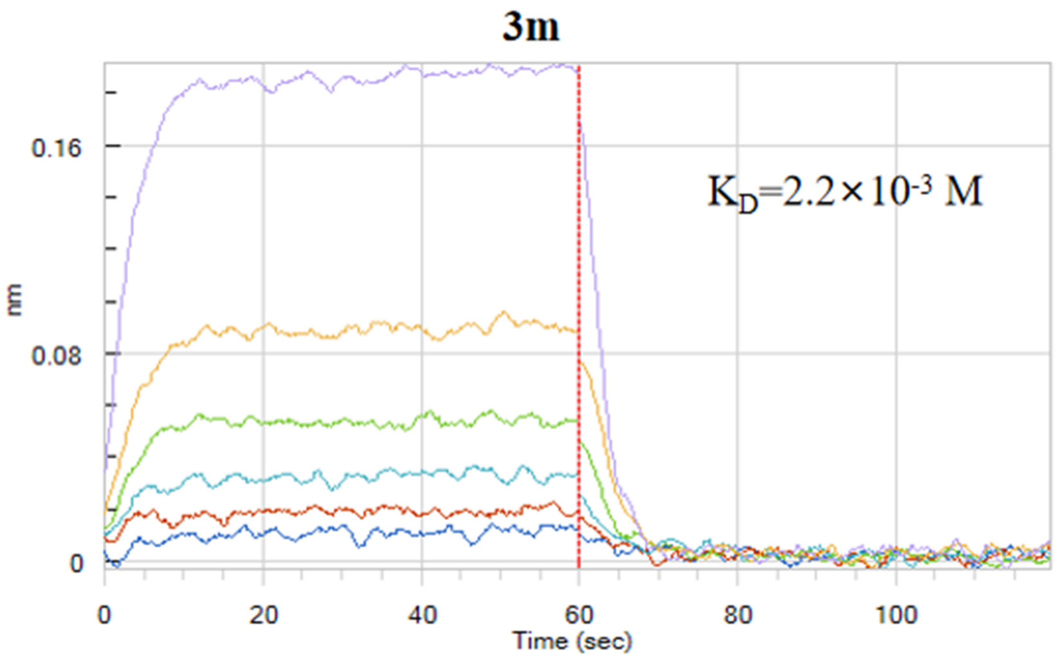

Figure S29. the affinity of compound **3m**

3n

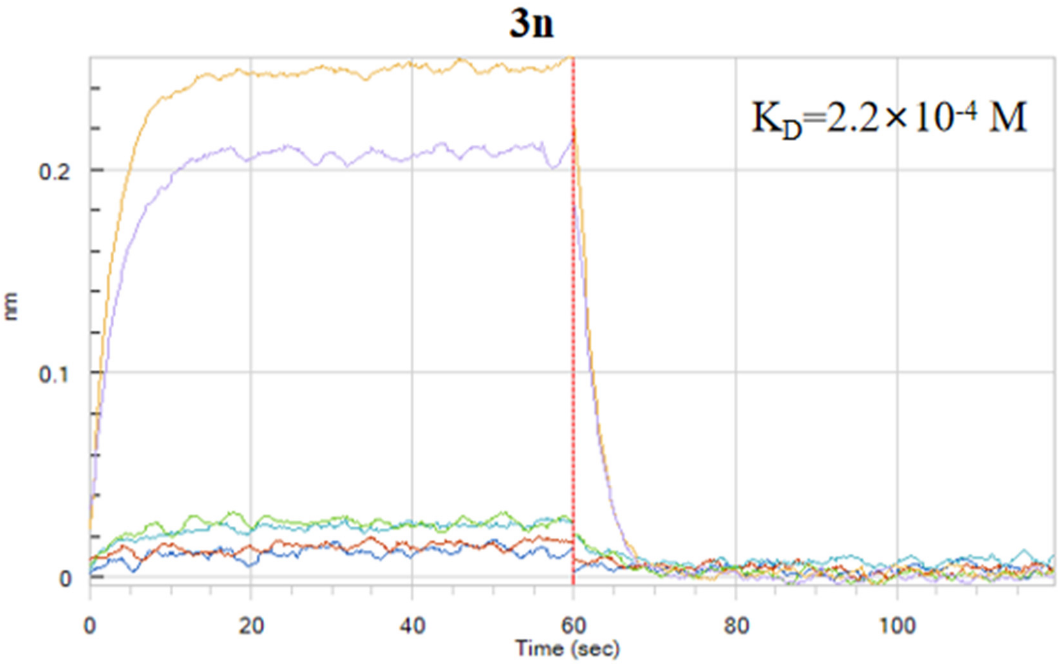

Figure S30. the affinity of compound **3n**

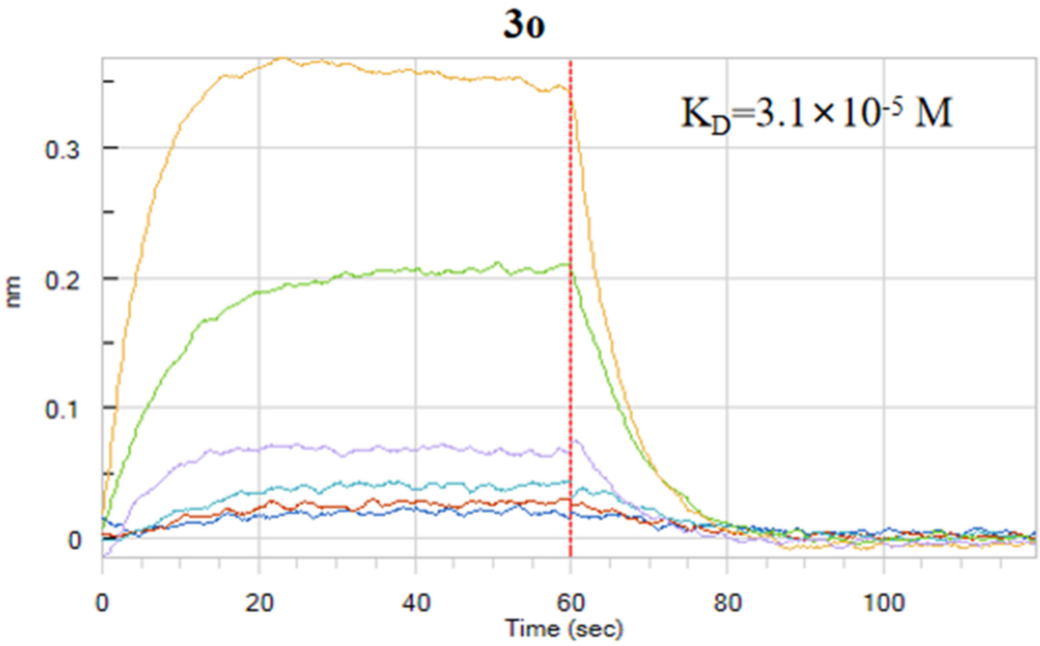

Figure S31. the affinity of compound **3o**

3p

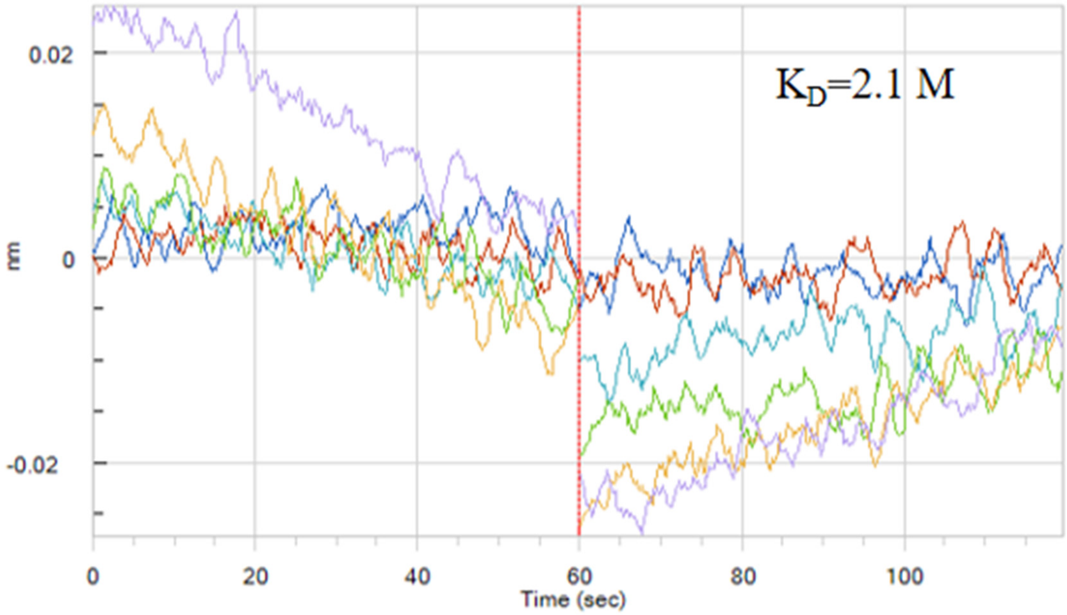

Figure S32. the affinity of compound **3p**

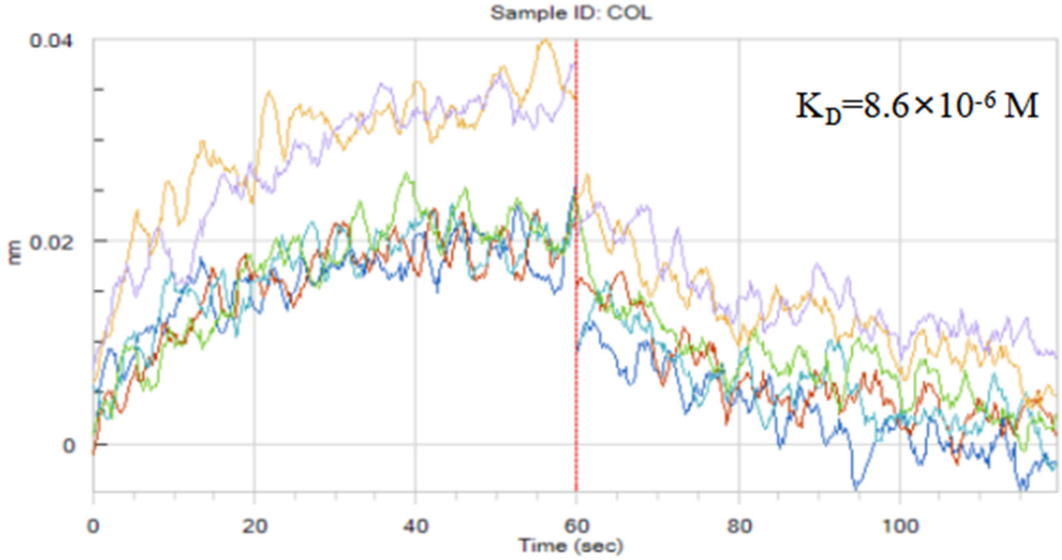

Figure S33. the affinity of colchicine

Docking Studies of the Compound 3g

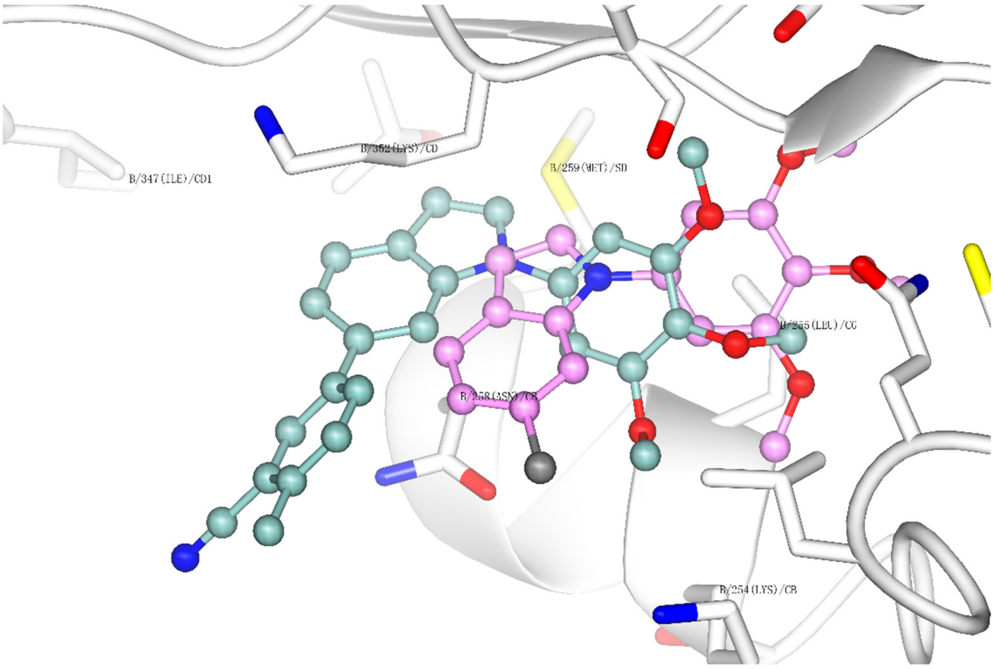

Figure S34. Superposition of the docking poses of 3g (sea green) and the original hit compound (pink) in the colchicine-binding site of tubulin. Docking was initialised using the document PDB 3E22 and achieved by MOE. Figures were imaged by CCP4MG.

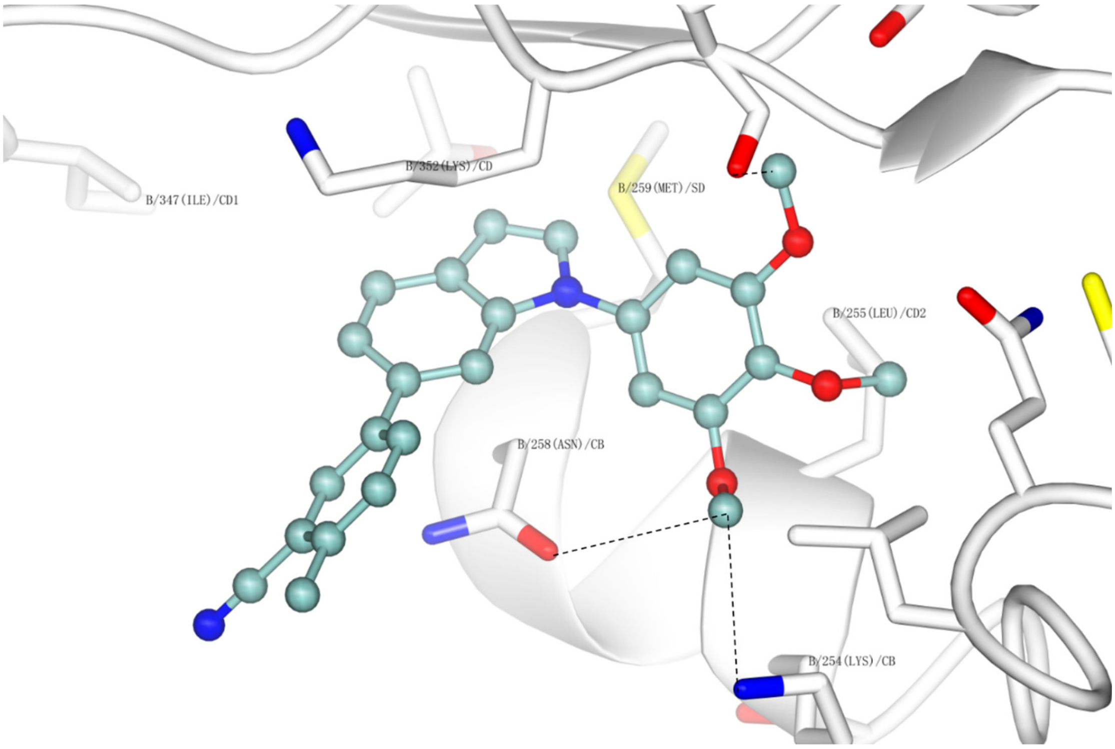

Figure S35. Predicted binding pose of compound 3g in the colchicine-binding site of tubulin. Docking was initialised using the document PDB 3E22 and achieved by MOE. Figures were imaged by CCP4MG.
